# Stability of Some Ternary 13-Atom Icosahedral Clusters Assessed with Geometric, Electronic, and Thermodynamic Criteria

**DOI:** 10.1021/acs.jpca.6c02945

**Published:** 2026-07-06

**Authors:** Anirudh Krishnadas, João Marcos Tomaz Palheta, Jonathan Bekele Mekonnen, Renato Luis Tame Parreira, Efracio Mamani Flores, René Fournier, Maurício Jeomar Piotrowski

**Affiliations:** † Department of Distributed Algorithms and Supercomputing, 39043 Zuse Institute Berlin, Berlin 14195, Germany; ‡ Department of Physics, 37902 Federal University of Pelotas, PO Box 354, Pelotas, Rio Grande do Sul 96010-900, Brazil; § Department of Chemistry, 7991 York University, Toronto, Ontario M3J 1P3, Canada; ∥ Núcleo de Pesquisas em Ciências Exatas e Tecnológicas, 92917 Universidade de Franca, Franca, São Paulo 14404-600, Brazil; ⊥ Department of Physics, Jorge Basadre Grohmann National University, Tacna 23000, Peru

## Abstract

Superatoms are clusters with physical or chemical properties that bear certain similarities to atoms. We calculated equilibrium geometries and properties using spin-polarized density functional theory (DFT) within the generalized gradient approximation (PBE). We also performed *Ab Initio* Molecular Dynamics (AIMD) simulations in the NVT ensemble with a Nosé-Hoover thermostat. In addition, Monte Carlo simulations with a potential fitted to DFT were carried out to assess a number of clusters as possible superatoms. The clusters we studied have a chemical composition AB_2_C_10_, where A, B, and C are metals, and a structure derived from that of a 13-atom icosahedron. We adopt an operational definition of superatom to assess clusters. According to this definition, superatoms should have (i) a quasispherical equilibrium geometry and (ii) a set of valence orbitals delocalized over the cluster; (iii) they should maintain these two attributes at elevated temperatures (mechanical and thermal stability); and (iv) they should be thermodynamically favored relative to similar clusters. By these criteria, Sn_3_Y_10_, CrSn_2_Zr_10_, and possibly CrSn_2_Ti_10_ can be classified as “superatoms”. We show that cluster stability arises from (i) the intrinsic stability of the *C*_10_ fragment; (ii) its interaction energy with the three “dopant atoms”; and (iii) compatible atomic sizes of the three elements. Clusters such as CrSn_2_Ti_10_ and CrSn_2_Zr_10_ exhibit an optimal balance among these contributions. They have a high effective coordination, an absence of very low vibrational frequencies, a favorable ratio of their elements’ cohesive energy, and exceptional thermal resilience, with melting temperatures that are well above the weighted average of their elements’ bulk melting points. Electronic analysis shows that CrSn_2_Ti_10_ and CrSn_2_Zr_10_ display discrete density-of-states features and partial electron delocalization, consistent with superatomic behavior. These electronic properties vanish as the clusters approach the melting point and undergo structural distortion. We show the importance of looking at several, often interrelated, properties in assessing possible superatoms.

## Graphical abstract

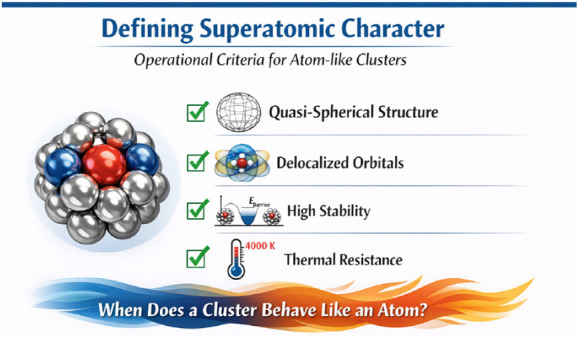

## 1 Introduction

Metal clusters with fewer than a hundred atoms are in a regime of matter where size, composition, and geometry all affect physical and chemical properties.^1–3^ In this regime, simple scaling laws often fail, and even the addition or substitution of a single atom can lead to significant, often counterintuitive, changes in stability, electronic structure, and chemical reactivity.^4,5^ These pronounced finite-size effects make nanoclusters attractive in areas such as catalysis, nanoelectronics, and energy-related applications.^6^

Thirteen-atom clusters, in particular, have attracted special attention because of the possibility of achieving closed atomic shells in icosahedral (ICO) geometries.^7,8^ This highly symmetric, close-packed structure minimizes surface energy and frequently corresponds to the global minimum for 13-atom elemental and doped clusters.^9,10^ Some of them also have a closed spherical electronic shell: they are “doubly magic” clusters.^2,11,12^ In addition to having a quasispherical atomic and electronic structure, some clusters have magnetic and chemical properties that make them similar to atoms and are called superatoms.^4,13^

Superatoms are generally understood as clusters of atoms that exhibit some physical or chemical properties similar to those of an atom. For instance, Al_13_ is considered a superhalogen.^14^ Its valence electron count, 39, is one short of achieving a closed electronic shell. It has a large electron affinity (3.57 eV)^15^ and ${\mathrm{Al}}_{13}^{-}$ binds to an iodine atom without changing its shape or charge state.^14^ Comparing the chemistry of atoms to that of presumed superatoms is complicated because atoms can react in different ways with a multitude of reaction partners. Here, we choose to focus on atomic properties that have no equivalent in most molecules. (1) The nucleus of an atom of a stable isotope does not split or distort during a physical or chemical transformation. (2) Atoms have spherical potential, electron density, and electronic shells. (3) Atoms may lose or gain electrons or may bind to other atoms or molecules, but they retain their identity (they do not undergo transmutation). The operational definition of a superatom that we use here is that of a cluster with the same or similar qualities: (1) the cluster is mechanically stable and does not distort easily, (2) it has a quasispherical nuclear framework and electronic shells, (3–1) it keeps its quasispherical shape and remains solid-like up to high temperatures, and (3–2) it is thermodynamically stable relative to clusters with similar chemical compositions.

The possibility of tuning the cluster properties with controlled chemical substitution is compelling. First-principles calculations indicate that doping can significantly reshape the geometric and electronic structure of nanoclusters.^16,17^ Icosahedral cage-like clusters of the form *A*@*B*_12_ (*B* = Ti, Zr, or Hf) are particularly interesting, as they combine a robust geometric structure with electronic tunability since the choice of the central atom *A* affects the electron count and the filling of electronic shells.^17^ The stability of such clusters cannot be predicted with simple formulas: atomic sizes, cohesive energies, electronic shell closings, magnetism, electron delocalization, *d*-*d* bonding can all play a role and interact in complex ways.^13^ Indeed, energy decomposition analyses have shown that nonadditive contributions matter and that nanoclusters are intrinsically many-body systems.^3^

Most computational studies have focused on elemental or binary clusters using stability descriptors such as binding energy, HOMO–LUMO gap, or magnetic moment.^1,2,8^ These properties are important but give an incomplete view of stability and superatomic character. The presence of more than one element and the reduced symmetry (relative to the bulk) bring into play multiple factors: surface energy, local strain, coordination mismatch, hybridization, etc. These cannot be captured by a single descriptor.^3^ Rationalizing the stability of small binary and ternary clusters using a small set of physically meaningful descriptors remains a challenge. Artificial atoms and superatomic clusters have also been proposed as building blocks for cluster-assembled materials, where stable clusters retain their identity while forming condensed phases with novel electronic and structural properties.^18–20^ These studies show the need to identify thermodynamically and mechanically robust cluster motifs, as their stability is a prerequisite for the realization of cluster-based materials.

Here, we present a thorough first-principles investigation of ternary 13-atom clusters with general composition AB_2_C_10_. Our goal is to quantify different aspects of their stability and superatomic character and to identify the physical factors that help explain their stability. We combine several geometric and electronic descriptors: effective coordination numbers, bond-length distribution, energy decomposition into interaction and distortion contributions, vibrational modes as indicators of mechanical stability, electronic density of states and HOMO–LUMO gaps, electron localization function, and heat capacity and other temperature-dependent properties obtained with two independent simulation methods: *Ab Initio* Molecular Dynamics (AIMD) at the level of DFT and Parallel Tempering Monte Carlo (PTMC) with an accurate graph neural-network potential trained on DFT energies. Taken together, these results show that stability can be understood on the basis of three things: (i) the intrinsic stability of the C_10_ subcluster, (ii) the interaction strength between host and dopant atoms, and (iii) the structural distortion required to fit the linear AB_2_ in the C_10_ fragment. We also find that the electronic qualities of a superatom are lost near the melting point when the cluster distorts. More broadly, the criteria and descriptors introduced here can define a practical and transferable framework for identifying superatomic behavior in complex nanoclusters, which can be applied beyond the specific systems studied in this work.

## 2 Methodology and Computational Details

One set of DFT calculations was performed within spin-polarized scalar-relativistic calculations^21,22^ using the generalized gradient approximation for the exchange-correlation functional with the Perdew–Burke–Ernzerhof^23^ (PBE) parametrization and the Vienna *Ab-initio* Simulation Package (VASP).^24–26^ The Kohn–Sham (KS) equations are solved using the all-electron projector augmented wave method,^27,28^ expanding the KS orbitals in plane waves up to a cutoff energy of 500 eV, corresponding to a value 20% larger than the VASP-recommended cutoff (ENMAX). For electronic structure analysis, the cutoff was further increased by 20%. Clusters were simulated in a large cubic supercell of size 20 Å, avoiding interactions with periodic images (since the distance between them is at least 14 Å), while free atoms are simulated in an orthorhombic supercell of size 19 Å × 19.25 Å × 19.5 Å. Since there is no electronic dispersion in isolated clusters, the Brillouin zone (BZ) integration was performed with a single **k**-point, i.e., the Γ-point. From convergence tests, the ternary cluster equilibrium geometries were obtained until atomic forces on every atom were smaller than 0.015 eV/Å, and a total energy convergence of 1.0 × 10^–6^ eV was achieved.

This same DFT framework was used to investigate finite-temperature properties via *Ab Initio* Molecular Dynamics (AIMD) simulations in the NVT ensemble using a Nosé-Hoover thermostat^29,30^ for thermalization (at 300 K) and annealing (up to 5000 K). Heating and cooling protocols were applied to extract melting behavior and structural resilience. Our AIMD simulations were run for 10 and 20 ps, respectively, with a time step of 1 fs. Note that in small clusters simulated under the NVT ensemble, the instantaneous temperature *T*(*t*) derived from the ionic kinetic energy exhibits statistical fluctuations around the target thermostat value. These fluctuations arise from the limited number of degrees of freedom and are expected even in equilibrium conditions. In addition, it is important to note that the same AIMD protocol was consistently applied to all clusters to ensure comparability of the results. Therefore, differences in melting behavior arise from intrinsic energetic and structural properties rather than from variations in simulation parameters.

A second set of DFT calculations was performed using the Gaussian software package using the meta-GGA approximation of Tao, Perdew, Staroverov, and Scuseria (TPSS)^31^ or the ωB97XD approximation for exchange and correlation, with D95 basis sets and SDD pseudopotentials.^32,33^ This was the methodology used in screening possible superatoms, as described in the next paragraph, and for constructing Machine Learning Interatomic Potentials (MLIPs) for use in Parallel Tempering Monte Carlo (PTMC) simulations.

We used simple criteria and electronic structure calculations to screen many 13-atom icosahedral clusters with singlet ground states and composition AB_2_C_10_ (atom A is at the center of the icosahedron, and the two atoms of B are opposite each other) for possible superatoms. We took every combination of “B” and “C” among these elements: Li, Be, Na, Mg, Al, K, Ca, Sc, Ti, Cu, Zn, Ga, Rb, Sr, Y, Zr, Ag, Cd, In, Sn, Au; and we took “A” among those same 21 elements plus Cr, Ru, and Pd. We imposed two constraints to eliminate combinations unlikely to produce stable clusters. For that, we define a nominal surface energy for an element as *E*_*s*_ = *E*_*c*_/4π*R* ^2^, where *E*_*c*_ and *R* are the cohesive energy and atomic radius. We kept only AB_2_C_10_ combinations where (a) the sum of atomic numbers is even, and (b) *E*_*s*_(A) ≥ *E*_*s*_(B) and *E*_*s*_(A) ≥ *E*_*s*_(C). This gave 2528 clusters. From those, we picked a pseudorandom subset of 192 that is diverse in terms of chemical composition and number of valence electrons *N*_*e*_ and carried out geometry optimizations and frequency calculations with the ωB97XD functional. The 192 atomization energies *AE* (defined here as positive numbers) were fitted to a linear regression model with 24 adjustable parameters *e*_*i*_:

$$AE_{j}=\underset{i}{\sum }e_{i}n_{ij}+r_{j}$$

Here, *n*_*ij*_ is the number of atoms of element *i* in cluster *j*, *e*_*i*_ is an effective cohesive energy for element *i*, and *r*_*j*_ is a residual, i.e., the part of the atomization energy of cluster *j* that is not explained by the model. We then selected eight clusters among the 192 as candidate superatoms on the basis of two criteria: a large positive *r*_*j*_ (indicative of thermodynamic stability) and a large HOMO–LUMO gap (indicative of chemical stability). They are CrLi_2_Ag_10_, CrSn_2_Ti_10_, CrSn_2_Zr_10_, Sn_3_Ca_10_, Sn_3_Y_10_, ZrAu_2_Rb_10_, ZrK_2_Cu_10_, ZrSr_2_Cu_10_.

We performed PTMC simulations for the candidate superatoms, each time with 35 replicas exploring a wide range of temperatures. Potential energies were calculated with an equivariant deep-learning interatomic potential (Allegro)^34^ that was fitted to DFT energies (obtained with the TPSS functional) of more than 2500 configurations. We trained and validated separate Allegro models for each superatom. The mean absolute error of the fit was less than 10 meV/atom in all cases considered. Heat capacities and other indicators of cluster melting were calculated as averages over a million or more PTMC simulation steps. The PTMC-DFT-Allegro methodology used here was described in detail in ref 35.

## 3 Results and Discussion

### 3.1 Structural and Dynamical Stability and Energetic Landscape

We begin by examining the structural stability of the ternary 13-atom clusters based on optimized geometries, binding energies, and effective coordination environments obtained with the VASP/PBE methodology. The optimized geometries of the ternary 13-atom clusters are shown in Figure 1a. Across the investigated compositions, the lowest-energy configuration maintains an icosahedral (ICO) shape, with some distortion, in which a central atom “A” is surrounded by ten atoms “C” forming a D_5*d*_ symmetry unit and capped at the poles by two atoms “B”. Despite this overall structural similarity, clear differences emerge in the degree of geometric distortion and energetic stabilization depending on chemical composition. CrSn_2_Ti_10_ and CrSn_2_Zr_10_ systems adopt a near-symmetric icosahedral structure where one Cr atom is centered and surrounded by a shell of Sn and Ti/Zr atoms. This central positioning of the smaller, higher-valent Cr atom may contribute to enhanced stability through strong directional bonding. On the other hand, some clusters, for instance, ZrK_2_Cu_10_ and ZrRb_2_Au_10_, deviate considerably from this structure, exhibiting stretched or less symmetric configurations that are related to lower effective coordination numbers (ECN) and reduced vibrational stability (ω_min_). The observed structural diversity underscores the impact of specific elemental composition and atomic size on the equilibrium geometry of ternary clusters.

**Figure 1:**
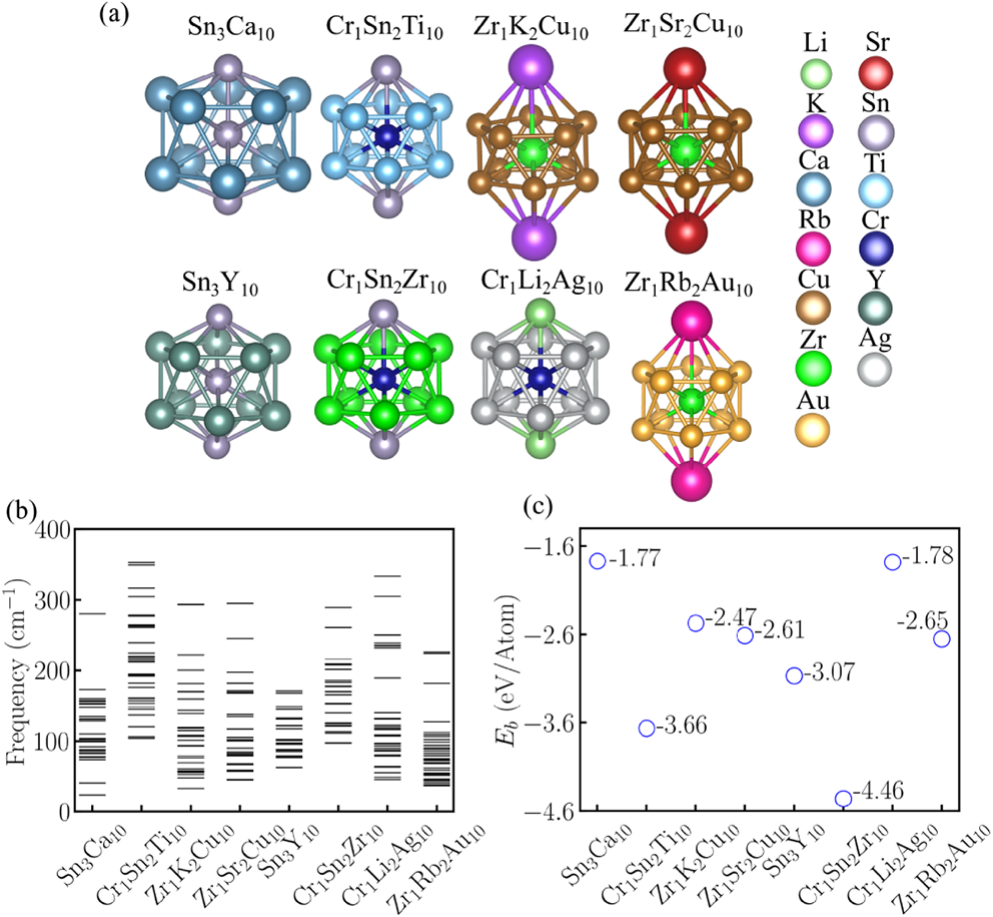
(a) Optimized geometric structures of the studied 13-atom ternary clusters. (b) Vibrational spectra of the studied clusters. The minimum vibrational frequency (ω_min_), for each system, is a direct measure of mechanical rigidity and resistance to distortion. (c) Average binding energy per atom (*E_b_*) for each ternary cluster. More negative values indicate stronger overall binding and greater thermodynamic stability.

The dynamical stability of the optimized structures is assessed through vibrational analysis, focusing on the minimum vibrational frequencies ω_min_, which are presented in Figure 1b. All stable configurations exhibit strictly positive (real) frequencies, confirming that they correspond to local minima on the potential energy surface, i.e., the vibrational spectra confirm mechanical stability for all clusters, with no imaginary frequencies. However, the magnitude of the lowest frequency of vibration, ω_min_, has large variations across the different systems, providing insight into their relative rigidity. Clusters with high structural stability are characterized by large ω_min_ values (more rigid structures and better distortion resistance), which indicate steep local curvature of the potential energy surface and resistance to atomic displacements. These systems are typically those with high ECN and low distortion energy, reinforcing the connection between geometric compactness and mechanical robustness. Consistent with their high-symmetry geometries, the CrSn_2_Ti_10_ and CrSn_2_Zr_10_ clusters possess the highest ω_min_ values (110 and 90 cm^–1^, respectively). These values are characteristic of quasi-icosahedral systems with strong interatomic bonding. On the other hand, clusters with lower ω_min_ values can show structural fluctuations, which reflects softer bonding environments and increased anharmonicity. For example, ZrK_2_Cu_10_ and ZrRb_2_Au_10_ clusters, with low values (25 cm^–1^), exhibit more flexible vibrational behavior and higher structural fragility, in agreement with observed geometric distortions.

We have quantified the thermodynamic stability of the clusters by calculating the average binding energy per atom (*E*_*b*_):

1 $$E_{b}=\frac{E_{\mathrm{tot}}^{\mathrm{cluster}}-E_{\mathrm{tot}}^{A}-2E_{\mathrm{tot}}^{B}-10E_{\mathrm{tot}}^{C}}{13}$$

where A, B, and C are the atomic species in the AB_2_C_10_ clusters. A more negative *E*_*b*_ means greater stability relative to the isolated constituent atoms. The *E*_*b*_ results are presented in Figure 1c, where they reveal a systematic dependence on the host subcluster nature and the incorporated dopants. Clusters based on Ti_10_ and Zr_10_ frameworks exhibit higher stability, indicating that these subclusters provide a robust structural backbone capable of accommodating chemical heterogeneity with minimal energetic penalty. The strong binding in CrSn_2_Ti_10_ and CrSn_2_Zr_10_ correlates directly with their high structural symmetry, large ω_min_, and (as shown later) high melting points. Likewise, the smaller *E*_*b*_ values for ZrK_2_Cu_10_ and CrLi_2_Ag_10_ are consistent with their structural distortions and lower vibrational stability.

To rationalize these trends, we decompose the total binding energy into physically meaningful contributions, separating the interaction energy (*E*_int_) between fragments, from the distortion energy (Δ*E*_dist_) required to accommodate the final geometry, and from the binding energy of the pristine 10-atom subcluster $\left(E_{b}^{u}\right)$ , as shown in Figure 2a and in Supporting Information. The binding energy, rewritten as $E_{b}=\left(E_{\mathrm{int}}+10\Delta E_{\mathrm{dist}}+10E_{b}^{u}\right)/13$ , reveals that highly stable clusters minimize the structural distortion energy while maintaining strong interatomic interactions. The most stable clusters combine a high intrinsic stability of the C_10_ subcluster and dopant atoms AB_2_ that do not significantly disrupt the local coordination environment.

**Figure 2:**
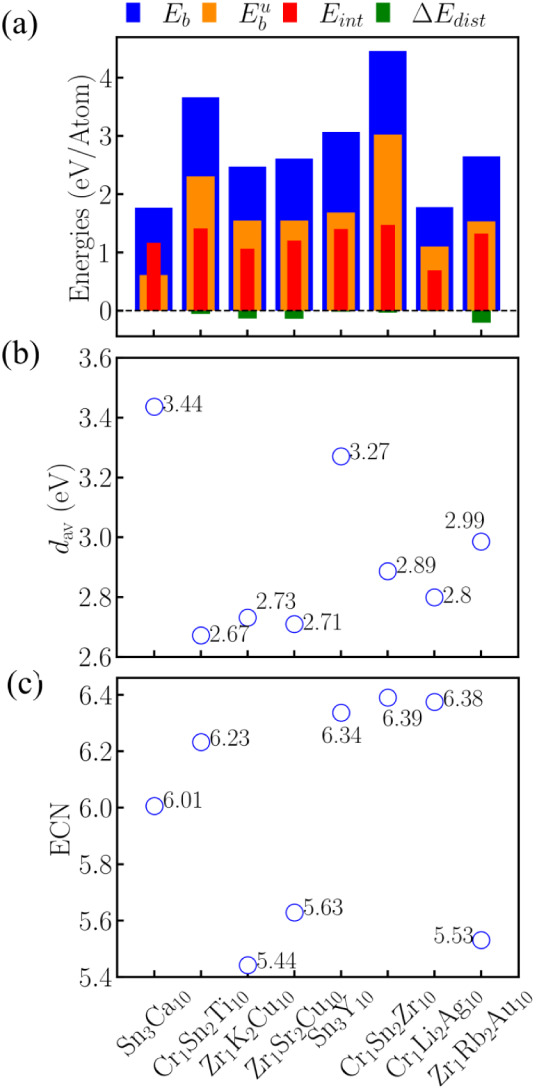
(a) Decomposition of the binding energy into interaction energy (*E*_int_), distortion energy (Δ*E*_dist_), and the binding energy of the 10-atom subcluster $\left(E_{b}^{u}\right)$ . (b) Average bond length (*d*_av_) and (c) effective coordination number (ECN) for the studied clusters. The ideal ECN (≈ 6.46) is given for a perfect ICO.

The energy decomposition also provides a measure of the energetic cost of accommodating the dopant atoms in the icosahedral framework. Clusters with small distortion energies can maintain good bonding interactions without suffering a large penalty for structural strain. Large distortion costs negate otherwise-favorable interaction energies.

In the most stable clusters, CrSn_2_Ti_10_ and CrSn_2_Zr_10_, the stability is primarily driven by two factors: (*i*) the inherent stability of the Ti_10_ or Zr_10_ subcluster itself (large, negative $E_{b}^{u}$ ), and (*ii*) a favorable interaction energy (*E*_int_) between this subcluster and the dopant (Cr, Sn) atoms. The distortion penalty (Δ*E*_dist_) is minimal for these systems, indicating that the optimal geometry of C_10_ is well-suited to accommodate the dopants within an ICO framework. In less stable clusters, a significantly positive Δ*E*_dist_ offsets the gains from *E*_int_. The structural compatibility between atomic species clearly plays a role. Clusters with large distortion penalties exhibit weakened effective bonding and reduced stability, even when the interaction energy remains favorable.

To quantify the structural integrity of the clusters, we analyze the ECN and the average bond length (*d*_av_) for all compositions (see Supporting Information), both presented in Figure 2b and c, respectively. ECN acts as a sensitive descriptor for local packing efficiency, which helps to capture deviations from ideal ICO coordination. High ECN values are consistently associated with configurations that preserve compact geometries and maximize atomic connectivity, while lower values indicate significant deviations from optimal coordination. The ECN of an ideal 13-atom ICO is a useful reference: it is 6.46.^9^

We see that the highest ECN values are associated with minimal structural distortion (minimal variation in *d*_av_), in agreement with the energetic analysis. In high ECN clusters, the atomic arrangement remains close to the ideal ICO geometry, leading to uniform bond distributions and reduced internal strain. The clusters with ECN greater than 6.2 are CrSn_2_Zr_10_ (6.39), CrSn_2_Ti_10_ (6.23), Sn_3_Y_10_ (6.34). The structural coherence in these clusters is also reflected in the small *d*_av_ variations, indicating homogeneous bonding environments. In contrast, clusters with lower ECN values, like ZrK_2_Cu_10_ (5.44) and ZrRb_2_Au_10_ (5.53), show significant deviations from ideal coordination, frequently characterized by elongated bonds and asymmetric atomic arrangements. These distortions reveal internal stress that contributes to the increase in distortion energy. The strong correlation between ECN and energy confirms the importance of geometric compatibility for stability. The ECN also correlates very well with ω_min_ and thermal resilience. A high ECN indicates a deep potential well, high rigidity, and stability.

### 3.2 Electronic Structure and Bonding Characteristics

The electronic properties of the clusters are investigated through the analysis of the HOMO–LUMO energy gaps, density of states (DOS), and electron localization function (ELF). The HOMO–LUMO gaps and representative DOS and ELF profiles are shown in Figure 3 (for Sn_3_Ca_10_, CrSn_2_Ti_10_, CrSn_2_Zr_10_, and ZrRb_2_Au_10_), while the DOS and ELF profiles for all clusters are summarized in Supporting Information (Figures S1 and S2). The HOMO–LUMO gap is an important electronic descriptor, indicative of chemical stability, electronic shell closure, and resistance to charge transfer. The PBE functional underestimates the absolute values of the gaps but provides reliable trends. Several clusters display relatively large HOMO–LUMO gaps from Figure 3a, suggesting enhanced electronic stability. However, a large gap does not imply structural stability. Indeed, some of the clusters with sizable gaps exhibit low structural stability as indicated by low ECN, low ω_*min*_, and large *d*_*av*_.

**Figure 3:**
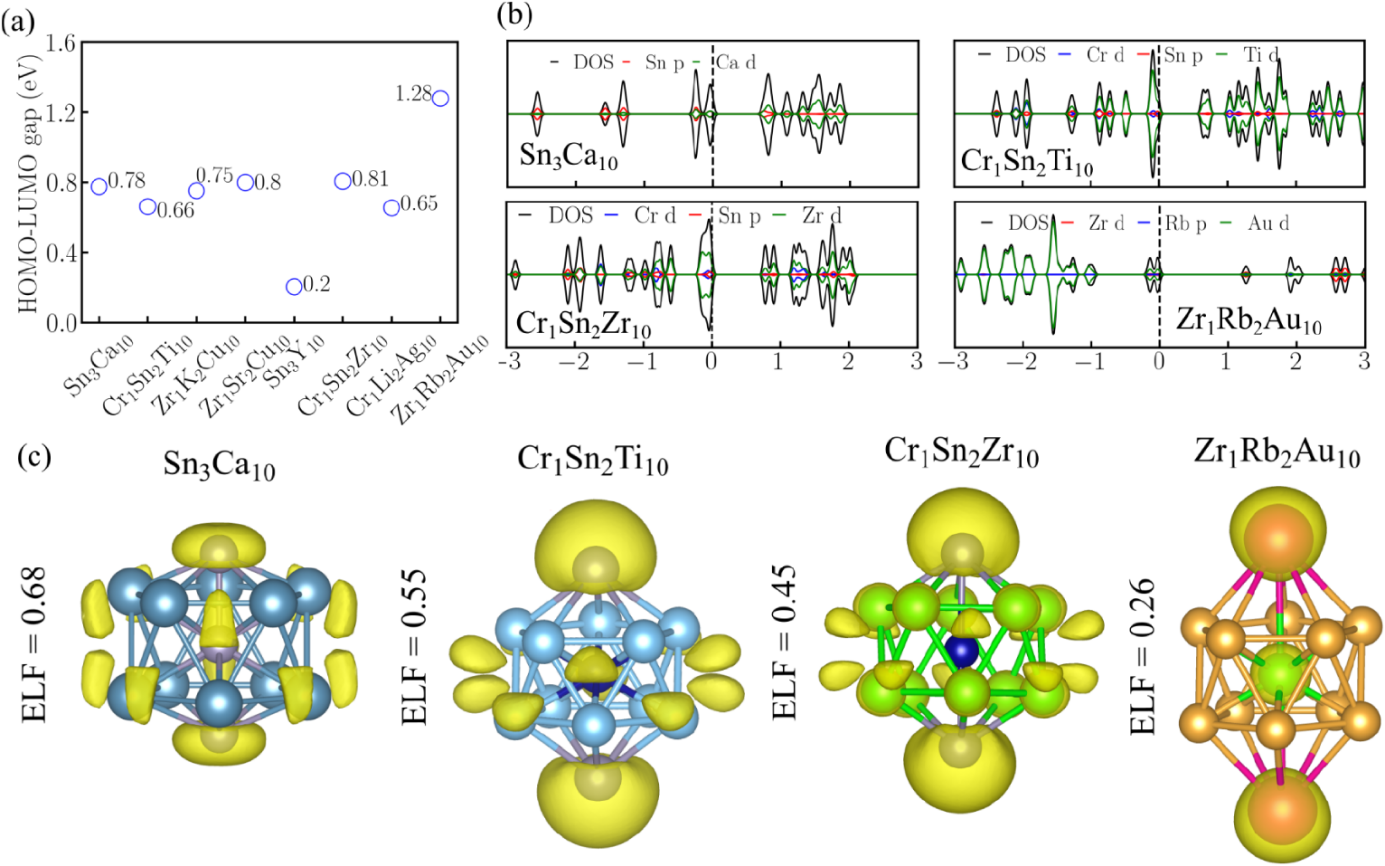
(a) HOMO–LUMO gap for all studied clusters. (b) Density of States (DOS) and (c) the electron localization function (ELF) for selected clusters (Sn_3_Ca_10_, CrSn_2_Ti_10_, CrSn_2_Zr_10_, and ZrRb_2_Au_10_). The Fermi level is presented by a dashed line at 0 eV. The DOS and ELF profiles for all clusters are presented in Supporting Information.

The clusters CrSn_2_Zr_10_ (0.81 eV) and CrSn_2_Ti_10_ (0.66 eV) exhibit moderate HOMO–LUMO gaps, consistent with electronically stable, closed-shell configurations expected for superatomic clusters. In contrast, Sn_3_Y_10_ possesses a very small gap (0.20 eV), suggesting electronic softness and low chemical stability. ZrRb_2_Au_10_ presents an interesting case. It has a large gap, 1.28 eV. However, as we will show from AIMD simulations, this is associated with a structural rearrangement toward a more symmetric D_2*d*_ motif upon annealing, aligning with the maximum hardness principle (MHP).

As we anticipated for finite systems, the computed DOS profiles have discrete energy levels, and the underlying atomic composition is reflected in the variations in peak distribution (see Figure 3b and Supporting Information). In particular, the DOS plots for CrSn_2_Ti_10_ and CrSn_2_Zr_10_ exhibit distinct peaks separated by gaps, especially near the Fermi level. The near degeneracies of energy levels giving rise to those peaks are characteristic of superatoms. These features are consistent with shell-like electronic structures typically associated with superatoms. The ELF analysis (Figure 3c and Supporting Information) provides additional insight. It shows the coexistence of partial electron delocalization and localized interaction regions. ELF maps allow one to describe bonds as predominantly metallic (delocalized) or covalent (localized). In the most stable clusters, we observed no indication of strongly localized states that would destabilize the framework. In clusters with higher ELF delocalization and smoother DOS profiles near the Fermi level, metallic bonding dominates and imparts a structural rigidity. Increased localization around certain sites can indicate directional bonding and also contribute to stability. The degree of delocalization is sensitive to the symmetry of the nuclear framework, as indicated by the ECN and *d*_*av*_.

We also optimized geometries and calculated equilibrium properties at the TPSS/SDD level with the Gaussian16 software package.^36^ These results are summarized in Table 1. Normal mode wavenumbers are reported for the lowest frequency, ω_*min*_, and for the seventh lowest frequency, ω_7_, as simple indicators of rigidity and mechanical stability. The rationale for looking at the seventh lowest frequency is that many clusters have two atoms of an element B with low cohesive energy, and, to a first approximation, these two atoms can account for up to six low-frequency modes. Hence, ω_7_ serves as a rough measure for the rigidity of the rest of the cluster. The value of *E*_*c*,*bulk*_ is calculated by taking one-thirteenth of the sum of the cohesive energies of elements A, B, and C, multiplied by their stoichiometric coefficients (1, 2, and 10, respectively).

**Table 1:** TPSS/SDD Properties at the Global Minimum Geometries of the Eight Clusters: Atomization Energies $U_{DFT}^{opt}$ , HOMO–LUMO gaps , Indicators of Mechanical Stability *ω_min_* and *ω_n_*, and Cluster Cohesive Energy Divided by a Weighted Average of the elements’ Bulk Cohesive Energies

	$U_{DFT}^{opt}\left(eV\right)$	HOMO–LUMO Gap (*eV*)	ω_*min*_ (*cm* ^–1^)	ω_7_ (*cm* ^–1^)	$\frac{E_{c,cluster}}{E_{c,bulk}}$
Sn_3_Y_10_	–42.76	0.23	38	83	0.80
CrLi_2_Ag_10_	–22.69	0.91	51	86	0.62
CrSn_2_Zr_10_	–55.31	0.83	99	125	0.76
CrSn_2_Ti_10_	–41.29	0.70	104	155	0.70
Sn_3_Ca_10_	–21.64	0.08	55	160	0.78
ZrRb_2_Au_10_	–34.83	1.16	24	35	0.76
ZrK_2_Cu_10_	–31.31	0.32	41	79	0.73
ZrSr_2_Cu_10_	–33.51	1.06	42	79	0.75

For metal clusters of this size, a HOMO–LUMO gap of 1 eV would be considered sizable, and 2 eV, very big. The HOMO–LUMO gaps calculated here are not remarkable. All ω_*min*_ values are large enough to confirm that the geometries are minima. The large ω_*min*_ of CrSn_2_Zr_10_ and CrSn_2_Ti_10_ suggest mechanical stability. The values of ω_7_ suggest that the C_10_ fragment is fairly rigid in all clusters except ZrRb_2_Au_10_. Small clusters have a ratio *E*_*c*,*cluster*_/*E*_*c*,*bulk*_ always smaller than 1 because of their large surface energy. For 13-atom metal clusters and their cations, this ratio is typically between 0.54 and 0.70,^12,37–39^ and a generic model of spherical metal clusters predicts it to be 0.72.^12^ By this measure, the last column of Table 1 suggests unusually strong bonding in Sn_3_Y_10_, CrSn_2_Zr_10_, Sn_3_Ca_10_, ZrRb_2_Au_10_, and ZrSr_2_Cu_10_.

### 3.3 Finite-Temperature Stability and Dynamical Behavior

To probe dynamic stability under realistic conditions, we conducted AIMD simulations. First, clusters were thermalized at 300 K for 10 ps (NVT ensemble). The temporal evolution of the total energy serves as a proxy for structural integrity. It is shown in Figure 4 for Sn_3_Ca_10_, CrSn_2_Ti_10_, CrSn_2_Zr_10_, and ZrRb_2_Au_10_, while the thermalization for all clusters is presented in the Supporting Information (Figure S3). Oscillations in total energy Δ*E*_tot_(*t*) indicate harmonic vibrations around a well-defined minimum, while smoother changes indicate nonrigidity. Most clusters, particularly CrSn_2_Ti_10_ and CrSn_2_Zr_10_, exhibit stable energy fluctuations around a well-defined mean at room temperature. A comparison of initial and final structures shows that most clusters retain their initial icosahedral shape. The most significant rearrangement occurred in ZrRb_2_Au_10_ (see Figure S3). It went from a low-symmetry local minimum to a higher symmetry (roughly D_2*d*_) geometry with a cube-like Au_10_ core. This new isomer exhibited a slightly larger HOMO–LUMO gap (1.34 eV *vs.* 1.28 eV), consistent with the MHP, which predicts that more stable isomers tend to maximize the HOMO–LUMO gap.

**Figure 4:**
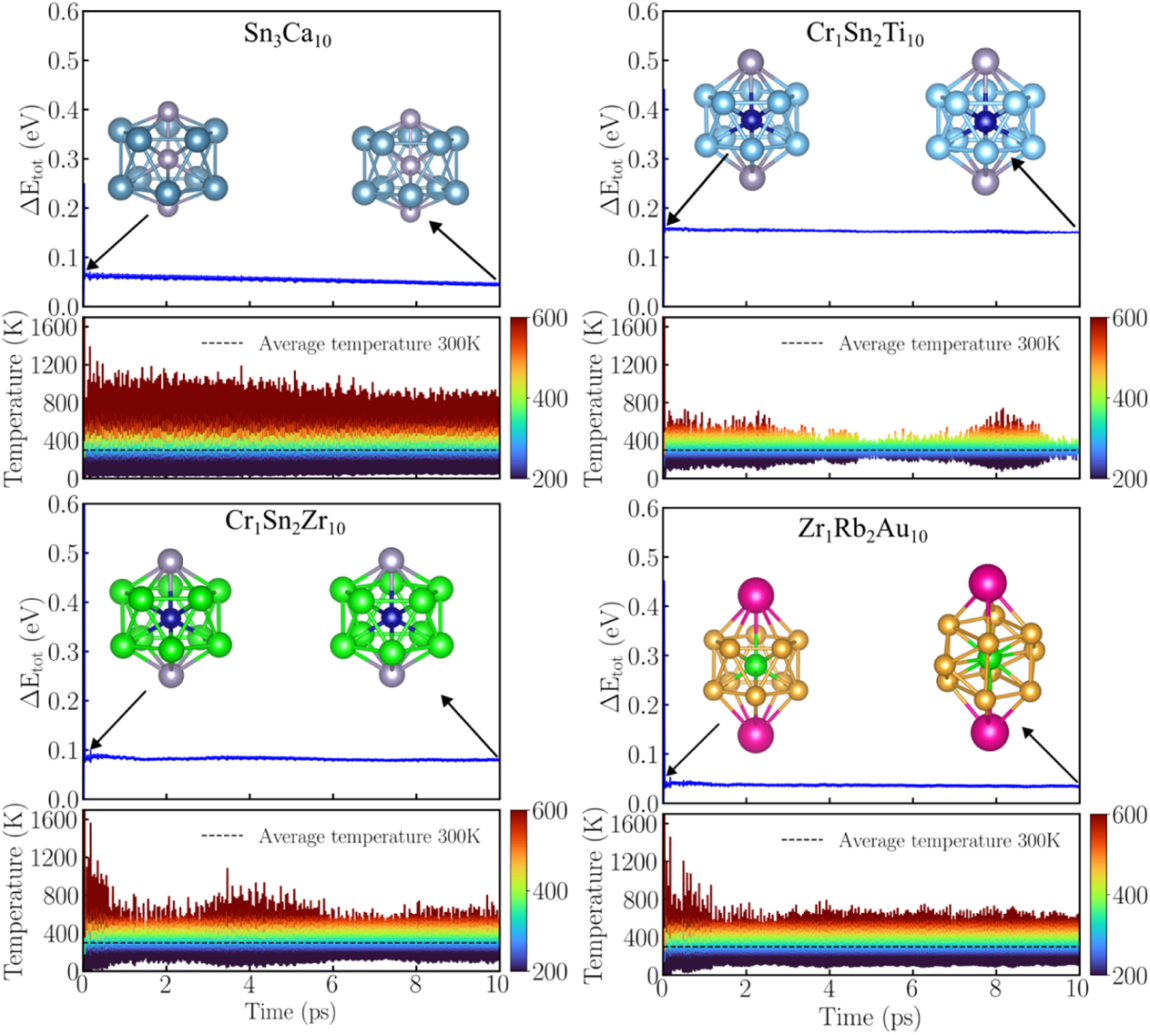
Thermalization at 300 K over 10 ps for Sn_3_Ca_10_, CrSn_2_Ti_10_, CrSn_2_Zr_10_, and ZrRb_2_Au_10_. The stability of the total energy (blue line) serves as a proxy for structural integrity under ambient conditions. Initial and final geometries are shown. The color scale represents the instantaneous ionic temperature *T*(*t*), which fluctuates around the thermostat set point due to finite-size and dynamical effects inherent to small clusters.

To test the thermal resilience, we performed AIMD simulations, heating the clusters from 0 to 5000 K over 20 ps. The evolution of the relative total energy as well as ECN and *d*_av_ with temperature is shown in Figure 5a and b for the most stable clusters, CrSn_2_Ti_10_ and CrSn_2_Zr_10_. Results for all clusters are summarized in the Supporting Information (Figures S4 and S5). In Figure 5a, we see well-defined oscillations in energy and ECN up to high temperatures, which indicate the preservation of structural integrity. On the contrary, less stable systems (see Supporting Information, Figure S4) show an early onset drift in energy and ECN, indicating greater distortions with increasing temperature. This could indicate premelting or progressive softening phenomena rather than an abrupt transition. Different regimes could contribute to the changes that we see in descriptors with increasing temperature in the AIMD simulations: harmonic vibrations, anharmonic motions, structural rearrangements, and melting.

**Figure 5:**
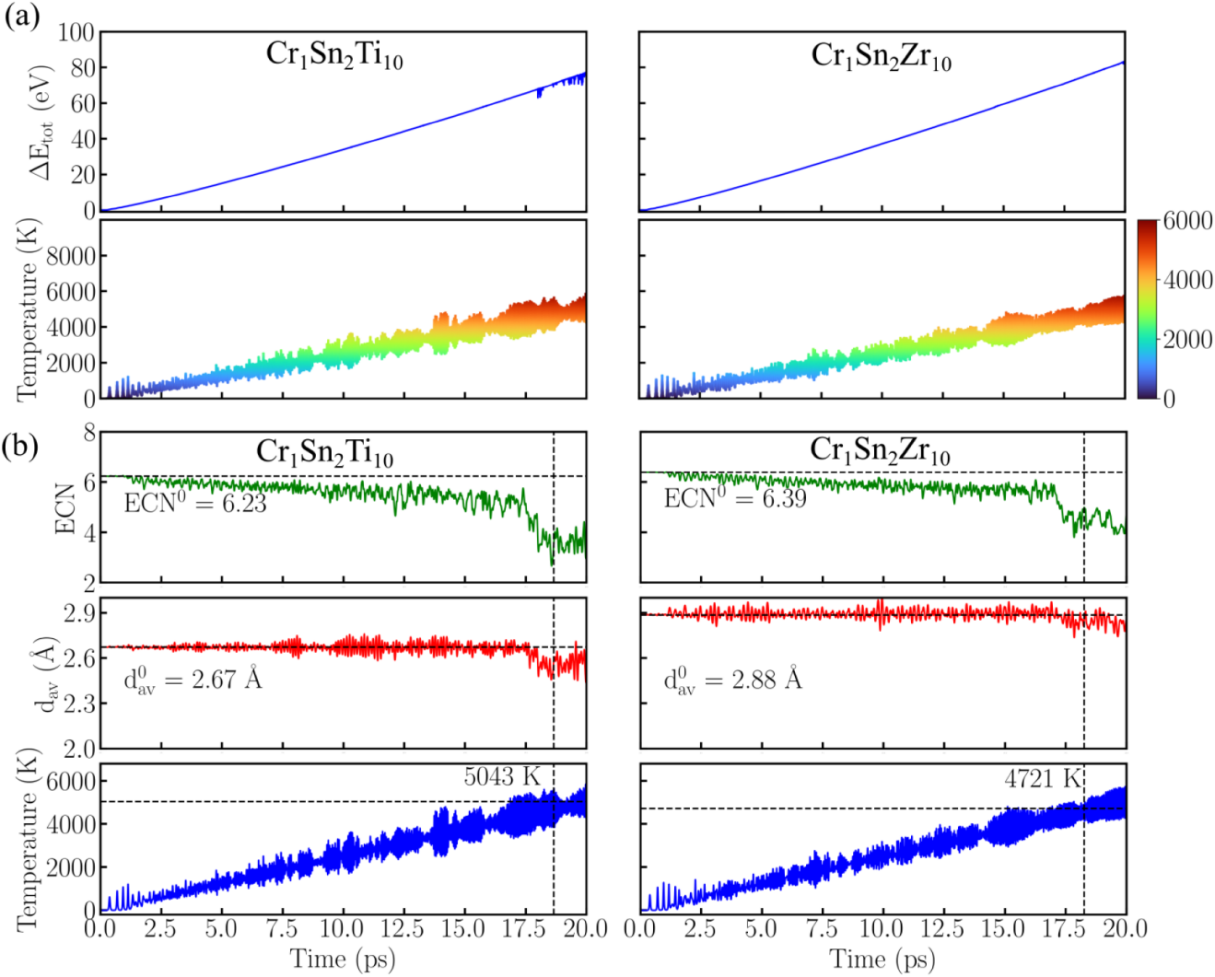
(a) Simulated melting dynamics or isomerization process: annealing from 0 to 5000 K over 20 ps. (b) Evolution of structural descriptors: ECN and *d*_av_, during heating, where the melting temperature (*T*_melting_) is identified as the point of abrupt change in these parameters, indicating loss of structural order. Both findings are presented for CrSn_2_Ti_10_ and CrSn_2_Zr_10_ clusters, while the Supporting Information shows the data for all clusters.

The cluster melting behaviors are shown in Figure 5b, where the most stable clusters present uncommonly high melting temperatures, consistent with their strong bonding and compact geometries. These results confirm that the descriptors identified at zero temperature, particularly ECN, distortion energy, and vibrational frequencies, are reliable predictors of finite-temperature stability. We estimated the melting point (*T*_melting_) by tracking the evolution of structural descriptors (ECN and *d*_av_) during heating. The point where these parameters deviate abruptly from their solid-state values marks the transition to a disordered liquid-like or isomerized state. The extracted melting temperatures are exceptionally high, with CrSn_2_Ti_10_ and CrSn_2_Zr_10_ leading at 5043 K and 4721 K, respectively. They greatly exceed the melting points of the corresponding metals in their bulk form. The melting temperatures obtained from the simulations should be regarded as upper bounds because the heating rate is high (275 K/ps), and there could be a time lag between the temperature and indicators like the ECN. The energy gain during heating for the most stable clusters is approximately 8.4 eV, consistent with the equipartition theorem expectation ( $\Delta E\approx \frac{3}{2}Nk_{B}T$ for a temperature of 5000 K).

We have also analyzed the instantaneous potential energy *U*(*t*)≡*E*_0_(*t*) (the electronic energy without the entropic smearing contribution, −*TS*, estimating the Born–Oppenheimer potential energy surface) along the AIMD trajectories (Figures S6–S9). Figure S6 shows that stable clusters, like CrSn_2_Ti_10_ and CrSn_2_Zr_10_, exhibit *E*_0_(*t*) traces with small-amplitude, high-frequency fluctuations around a steady mean, characteristic of atoms vibrating harmonically within a deep potential well. In contrast, less stable clusters show larger fluctuations, drifts in the baseline, and occasional sharp drops, signaling significant anharmonicity, isomerization events, or the onset of melting.

The energy autocorrelation function, ρ­(*k*), provides a quantitative measure of dynamical memory (see Supporting Information):

$$\rho \left(k\right)=\frac{{\langle [E_{0}\left(t\right)-{\mathrm{E̅}}_{0}]\left[E_{0}\left(t+k\right)-{\mathrm{E̅}}_{0}\right]\rangle }_{t}}{{\langle {[E_{0}\left(t\right)-{\mathrm{E̅}}_{0}]}^{2}\rangle }_{t}}$$

where *E̅*_0_ is the time-averaged potential energy and *k* is the discrete time lag between configurations. Its decay rate reveals the nature of atomic motion (Figure S7). A slow decay, as seen for CrSn_2_Ti_10_ and CrSn_2_Zr_10_, indicates strong correlation over time, typical of harmonic solids. As observed for ZrRb_2_Au_10_ and Sn_3_Ca_10_, a fast decay reflects the rapid loss of memory due to anharmonic effects, diffusion, or structural rearrangements associated with a liquid-like or soft solid state.

Figure S8 indicates that stable traces with low-amplitude fluctuations indicate mechanical robustness and preservation of the near-icosahedral geometry, whereas large excursions or baseline shifts signal structural rearrangements or the onset of melting. Finally, Figure S9 indicates that the clusters showing smooth and nearly constant energy evolution are mechanically stable, whereas those with pronounced energy fluctuations exhibit progressive softening or isomerization under heating. Stable clusters such as CrSn_2_Ti_10_ and CrSn_2_Zr_10_ exhibit small fluctuations around a well-defined mean value, indicative of strong interatomic interactions and a deep potential-energy minimum. In contrast, softer clusters (such as ZrRb_2_Au_10_) show larger oscillations and possible abrupt drops in *E*_0_(*t*), which correspond to isomerization events or partial structural rearrangements.

### 3.4 Interplay between Structural, Electronic, and Dynamical Stability

The combined analysis of structural, energetic, electronic, and dynamical properties reveals that cluster stability is inherently multidimensional. No single descriptor is sufficient to capture the full complexity of the stability landscape. Instead, stable clusters emerge from a cooperative interplay between geometric compactness (high ECN), favorable energetic balance (low distortion energy), and robust electronic structure. In particular, the results demonstrate that electronic features such as large HOMO–LUMO gaps or discrete DOS profiles are meaningful indicators of stability only when supported by a structurally coherent framework. Similarly, high coordination and low distortion energy are typically reinforced by favorable electronic interactions to ensure long-term dynamical stability at finite temperatures. This shows the usefulness of combining complementary descriptors for understanding or designing stable clusters.

All metrics show that the CrSn_2_Ti_10_ and CrSn_2_Zr_10_ clusters are stable, but the other clusters are susceptible to thermal excitation. The most stable clusters exhibit high thermal stability, which can be explained by structural and electronic factors. Local distortions under thermal excitation are lessened because the metallic cage’s near-ICO symmetry guarantees a uniform distribution of bonding interactions. Additionally, the bonding network is strengthened overall by the strong hybridization between the dopant atom and the host shell, which increases vibrational rigidity and, ultimately, melting temperatures. Superatomic stability is characterized by this synergistic effect between electronic delocalization and geometric symmetry.

### 3.5 Characterization of Melting Transitions by Parallel Tempering Simulations

Unlike bulk materials, clusters exhibit solid–liquid coexistence between *T*_*f*_ (freezing) and *T*_*m*_ (melting, *T*_*m*_ > *T*_*f*_) and display different signs of melting at different onset temperatures. We analyze PTMC simulations to determine *T*_*f*_ and *T*_*m*_ in two ways. First, we plot the width of the potential energy distribution *W*_*U*_ as a function of temperature and find the lower and upper temperatures (estimates of *T*_*f*_ and *T*_*m*_) where *W*_*U*_(*T*) deviates from a linear fit. Second, we train an Artificial Neural Network Classifier (ANNC) to distinguish low-temperature (*T* < *T*_*l*_) cluster configurations from high-temperature (*T* > *T*_*h*_) configurations and gradually increase *T*_*l*_ and decrease *T*_*f*_ until the ANNC makes mistakes in more than 5% of cases. The two limits serve as estimates for *T*_*f*_ and *T*_*m*_. We calculate the heat capacity *C*(*T*) with the energy fluctuation formula. The temperature at which *C*(*T*) is maximum is denoted *T*_*c*_. We calculate a dissimilarity index *D*(*X*_0_, *X*) between the global minimum and a configuration *X* by comparing their ordered sets of internuclear distances. The mean of *D*(*X*_0_, *X*_*j*_; *T*) for configurations *X*_*j*_ sampled at temperature *T*, δ­(*T*), increases with *T*. We use the maximum in the slope of δ­(*T*) to estimate *T*_*c*_. Finally, we calculate for a set of configurations sampled at temperature *T* the fraction *G* of internuclear distances that are inside a small interval centered at *d*_*m*_ = (*d*_1_ + *d*_2_)/2, where *d*_1_ and *d*_2_ are the positions of the first two peaks in the pair distribution function of the global minimum. A large *G* indicates that many atom pairs *ij* are neither nearest-neighbors (NN) nor next-nearest-neighbors (NNN), and a possible transition in their relative position from NN to NNN (or the reverse). We defined *T*_*n*_ as the temperature where the rate of increase in *G*(*T*) is maximum. The onset of the liquid phase and self-diffusion is associated with an increase in *G*. However, the reverse is not true: NN-to-NNN rearrangements may occur in a solid. We expect *T*_*n*_ ≤ *T*_*c*_. Details on how these properties and temperatures are obtained from PTMC simulations can be found in ref 40. The characteristic temperatures extracted from the PTMC simulations, including the transition temperatures obtained from heat-capacity, structural, machine-learning, and energy-distribution analyses, are summarized in Table 2.

**Table 2:** Temperatures *T_c_, T_n_, T_f_*, and *T_m_* (Kelvin) Indicative of Melting for Eight Clusters Obtained from Parallel Tempering Simulations by Calculating Tthe Heat Capacity *C*(*T*); an Index of Similarity to the Global Minimum Geometry (*δ*); a Count of Interatomic Distances Near *d_m_* = (*d*_1_ + *d*_2_)/2 (*G*); the Width of the Potential Energy Distribution (*W_U_*); and Solid and Liquid Labels Assigned to Configurations by an ANN Classifier^a^

	*T*_*c*_ (*C*(*T*), δ)	*T*_*n*_ (*G*)	*T*_*f*_ (ANNC, *W*_*U*_)	*T*_*m*_ (ANNC, *W*_*U*_)	*T*_*bulk*_
Sn_3_Y_10_	2000, 2250	2200	1150, 1300	2400, 2600	1500
CrLi_2_Ag_10_	1650, 1675	850	1300, 800	1800, 1800	1188
CrSn_2_Zr_10_	2500, 2875	2425	1350, 1750	2650, 2300	1882
CrSn_2_Ti_10_	1875, 1925	1950	1300, 1300	2250, 2300	1738
Sn_3_Ca_10_	950, 760	850	775, 650	1550, 1400	974
ZrRb_2_Au_10_	1400, 1200	1300	850, 600	1700, 1600	1240
ZrK_2_Cu_10_	900, 775	525	375, nm	900, nm	1259
ZrSr_2_Cu_10_	nm, 300	460	450, 600	1125, 1300	1368

a *T_bulk_* is a simple weighted average of experimental bulk melting points. nm: no indication of melting.

The two sets of *T*_*c*_ (obtained from *C*(*T*) and δ) agree with each other and so do the two sets of *T*_*m*_ (ANNC, *W*_*U*_). The agreement between the two sets of *T*_*f*_, and between *T*_*n*_ and *T*_*c*_, breaks down for CrLi_2_Ag_10_ and ZrK_2_Cu_10_. The low *T*_*n*_ in these clusters suggests that the increase in the number of atom pairs at distances intermediate between the first and second peaks of the pair distribution function is not due to melting; it is due to Ag–Ag and Cu–Cu atom pairs interchange at temperatures well below *T*_*f*_.

Leaving *T*_*n*_ aside, a comparison of *T*_*c*_, *T*_*f*_, and *T*_*m*_ to *T*_*bulk*_ (the average of experimental bulk melting points of the 13 atoms) shows that Sn_3_Y_10_, CrLi_2_Ag_10_, and CrSn_2_Zr_10_ have very high melting points, roughly 20% to 30% higher than the bulk. Clusters with fewer than 50 atoms typically melt at temperatures well *below* the bulk melting point. In that sense, these three clusters are unusual. CrSn_2_Zr_10_ has the highest absolute melting temperatures (*T*_*c*_, *T*_*f*_, *T*_*m*_), ∼2240 K. The relatively high HOMO–LUMO gap (0.83 eV) and smallest vibrational frequency (99 cm^–1^) also suggest that CrSn_2_Zr_10_ is stable. The melting indicators for CrSn_2_Zr_10_ are shown in Figure 6. All five criteria yield a consistent picture, a well-defined coexistence region and a sharp transition. Corresponding plots for the remaining seven clusters are provided in the Supporting Information. ZrK_2_Cu_10_ and ZrSr_2_Cu_10_, on the contrary, melt at very low temperatures relative to *T*_*bulk*_. A possible explanation for this is that having atoms of a low cohesive energy element at the poles of the ICO (Sr, K) destabilizes the remaining ten Cu atoms. However, Rb atoms in ZrRb_2_Au_10_ do not significantly destabilize the Au_10_ shell. The melting temperatures of CrSn_2_Ti_10_, Sn_3_Ca_10_, ZrRb_2_Au_10_ are unremarkable.

**Figure 6:**
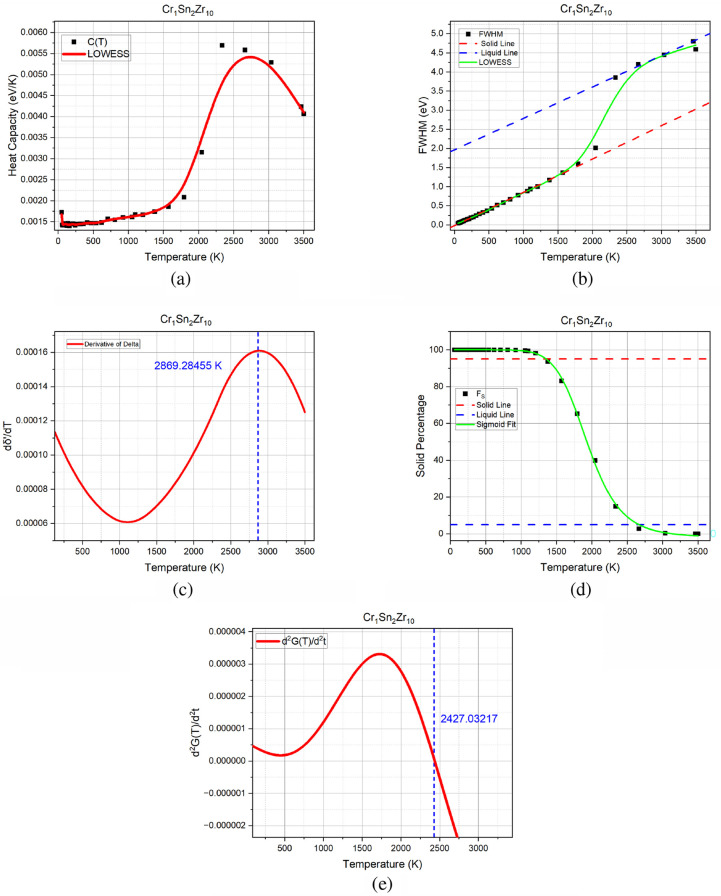
Temperature dependence of properties used to detect melting for CrSn_2_Zr_10_: *C*(*T*), *W_U_*, δ, ANNC *F_S_*, and inflection point *d* ^2^ *G*/*dT* ^2^. (a) Heat capacity curve (*C*(*T*)). (b) Width of the potential energy distribution (*W_U_*). (c) Derivative of δ with respect to temperature. (d) Configurations classified as solid (ANNC *F_S_*). (e) Second derivative of *G* with respect to temperature.

## 4 Conclusions

We used a few simple criteria derived from DFT-optimized equilibrium structures (an icosahedral shape, a relatively high index of thermodynamic stability, and a high HOMO–LUMO gap) to select eight clusters among 192 as potential superatoms. A detailed study of those eight clusters, looking at many more metrics and aspects of stability, showed that CrSn_2_Zr_10_ emerges as the most consistently stable system and a strong candidate for superatomic behavior. Other clusters stand out on some of the metrics: Sn_3_Y_10_ and CrSn_2_Ti_10_ have very high melting points; Sn_3_Y_10_ and Sn_3_Ca_10_ have unusually high atomization energies compared to their elements; CrSn_2_Ti_10_ is very rigid; and ZrRb_2_Au_10_ and ZrSr_2_Cu_10_ have sizable HOMO–LUMO gaps. More broadly, the framework proposed here provides a systematic route for identifying superatomic building blocks and may guide the rational design of cluster-based materials with atom-like functionalities.

We find that stability is determined by a complex interaction between geometric, energetic, and electronic factors. The most stable clusters generally have an ideal icosahedral shape, as indicated by a high effective coordination number (ECN ≳ 6.3) and small bond-length dispersion. They also show a good balance between strong interaction energies and low distortion. Size compatibility between dopants and the host subcluster is important. CrSn_2_Ti_10_ and CrSn_2_Zr_10_ are highly symmetrical and have a combination of mechanical stability (high ω_min_), favorable energetic contributions, well-defined discrete energy levels, and sizable HOMO–LUMO gaps that persist even at high temperatures. The electronic structure analysis indicates that superatom-like features, such as sharp peaks in the density of states and partial electron delocalization, are associated with these clusters. Surprisingly, the HOMO–LUMO gap is not a good predictor of stability. However, indicators of thermal stability obtained from simulations correlate quite well with other zero-Kelvin indicators of stability: a high ECN, low distortion energy, high ω_*min*_, and high *E*_*c*,*cluster*_/*E*_*c*,*bulk*_. By contrast, clusters with low ECN, ω_*min*_ and high distortion energy distort and melt at relatively low temperatures in our simulations.

Computing several physical properties and complementary indicators of stability of small binary and ternary clusters provides a systematic framework for identifying stable clusters and candidate superatoms and may guide the rational design of cluster-assembled nanomaterials.

## Supplementary Material



## Notes

Methodological details: energetic decomposition, effective coordination number concept, time-correlation and thermal analysis; density of states; electron localization function; thermalization – AIMD; melting behavior; potential energy estimation; melting indicators for individual clusters from parallel tempering simulations (PDF)
