# Supplementary material for: Stability of Some Ternary 13-Atom Icosahedral Clusters Assessed with Geometric, Electronic, and Thermodynamic Criteria
Source: J Phys Chem A. 2026 Jul 6;130(30):5852–62. doi: 10.1021/acs.jpca.6c02945 (PMC13430676; doi:10.1021/acs.jpca.6c02945)
Supplement: Supplementary file 1 [file jp6c02945_si_001.pdf]

Supporting Information: Stability of some Ternary 13-atom Icosahedral Clusters Assessed with Geometric, Electronic, and Thermodynamic Criteria

Anirudh Krishnadas,[†,⊥] João Marcos Tomaz Palheta,[‡,⊥] Jonathan Bekele Mekonnen,[¶] Renato Luis Tame Parreira,[§] Efracio Mamani Flores,[‖] René Fournier,[¶] and Maurício Jeomar Piotrowski[*,‡]

†*Department of Distributed Algorithms and Supercomputing, Zuse Institute Berlin, 14195 Berlin, Germany*

‡*Department of Physics, Federal University of Pelotas, PO Box* 354, *Pelotas, RS,* 96010-900, *Brazil*

¶*Department of Chemistry, York University, Toronto, ON M3J 1P3, Canada*

§*Núcleo de Pesquisas em Ciências Exatas e Tecnológicas, Universidade de Franca, Franca, SP,* 14404-600 *Brazil*

‖*Department of Physics, Jorge Basadre Grohmann National University, Tacna* 23000, *Peru*

⊥*These authors contributed equally to this work.*

E-mail: mauriciomjp@gmail.com

Contents

I Methodological Details **S-3**

A Energetic Decomposition S-3

B Effective Coordination Number Concept S-4

C Time-Correlation and Thermal Analysis S-4

II Density of States **S-6**

III Electron Localization Function **S-7**

IV Thermalization – AIMD **S-8**

V Melting Behavior **S-9**

VI Potential Energy Estimation **S-11**

VIIMelting Indicators for Individual Clusters from Parallel Tempering Simulations **S-15**

I Methodological Details

A Energetic Decomposition

To gain deeper insight into the factors governing cluster stability, we deconstructed the E_b into physically intuitive components: the interaction energy (E_{int}), the distortion energy (ΔE_{dist}), and the binding energy of the pristine 10-atom subcluster (E_b^u). The interaction energy,

$$E_{\text{int}} = E_{\text{tot}}^{\text{cluster}} - E_{\text{tot}}^{Z\text{–froz}} - E_{\text{tot}}^{X} - 2E_{\text{tot}}^{Y}, \tag{1}$$

measures the energy gain upon embedding the X and Y atoms into the frozen geometry of the Z_{10} subcluster. Here, $E_{\text{tot}}^{Z\text{–froz}}$ is the total energy of the optimized Z_{10} cluster held in its frozen geometry. The distortion energy,

$$\Delta E_{\text{dist}} = \frac{E_{\text{tot}}^{Z\text{–froz}} - E_{\text{tot}}^{Z\text{–opt}}}{10}, \tag{2}$$

quantifies the energy penalty required to distort the relaxed Z_{10} subcluster ($E_{\text{tot}}^{Z\text{–opt}}$) into the geometry it adopts within the ternary cluster. Finally, E_b^u is the binding energy per atom of the isolated, relaxed Z_{10} cluster:

$$E_b^u = \frac{E_{\text{tot}}^{Z\text{–opt}} - 10E_{\text{tot}}^{Z}}{10}. \tag{3}$$

The total E_b can be expressed as a combination of these terms:

$$E_b = \frac{E_{\text{int}} + 10\Delta E_{\text{dist}} + 10E_b^u}{13}. \tag{4}$$

Algebraic expansion confirms that Equation (4) is identical to the original definition of E_b used in the main manuscript, i.e.,

$$\begin{aligned}
E_b &= \frac{E_{tot}^{cluster} - E_{tot}^{Z-froz} - E_{tot}^{X} - 2E_{tot}^{Y} + 10\left(\frac{E_{tot}^{Z-froz} - E_{tot}^{Z-opt}}{10}\right) + 10\left(\frac{E_{tot}^{Z-opt} - 10E_{tot}^{Z}}{10}\right)}{13} \\
&= \frac{E_{tot}^{cluster} - \cancel{E_{tot}^{Z-froz}} - E_{tot}^{X} - 2E_{tot}^{Y} + \cancel{E_{tot}^{Z-froz}} - \cancel{E_{tot}^{Z-opt}} + \cancel{E_{tot}^{Z-opt}} - 10E_{tot}^{Z}}{13} \\
E_b &= \frac{E_{tot}^{cluster} - E_{tot}^{X} - 2E_{tot}^{Y} - 10E_{tot}^{Z}}{13}\,,
\end{aligned} \tag{5}$$

B Effective Coordination Number Concept

The Effective Coordination Number (ECN) provides a measure of local atomic environment and cluster compactness, generalizing the classical integer coordination number by employing a smooth weighting function based on interatomic distances:

$$\mathrm{ECN}_i = \sum_{j\neq i} \exp\left[1 - \left(\frac{d_{ij}}{d^i_{\mathrm{av}}}\right)^6\right]. \tag{6}$$

The weighting function ensures that atoms significantly closer than the average distance d^i_{av} contribute more strongly to the coordination. The average distance d^i_{av} for each atom i is determined self-consistently from:

$$d^i_{\mathrm{av}} = \frac{\sum_j d_{ij} \exp\left[1 - \left(\frac{d_{ij}}{d^i_{\mathrm{av}}}\right)^6\right]}{\sum_j \exp\left[1 - \left(\frac{d_{ij}}{d^i_{\mathrm{av}}}\right)^6\right]}. \tag{7}$$

The mean ECN and mean bond length (d_{av}) for the entire cluster are then given by:

$$\mathrm{ECN} = \frac{1}{N}\sum_{i=1}^{N} \mathrm{ECN}_i, \qquad d_{\mathrm{av}} = \frac{1}{N}\sum_{i=1}^{N} d^i_{\mathrm{av}}. \tag{8}$$

C Time-Correlation and Thermal Analysis

The instantaneous potential energy used in the dynamical analysis is the VASP-reported `E0` (electronic energy excluding the entropic smearing contribution), which represents the Born–Oppenheimer potential energy surface for the ionic coordinates:

$$U(t) \equiv E_0(t).$$

The energy autocorrelation function was computed in its normalized discrete form:

$$\rho(k) \;=\; \frac{\langle [U(t) - \overline{U}]\,[U(t+k) - \overline{U}]\rangle_t}{\langle [U(t) - \overline{U}]^2\rangle_t},$$

where $\overline{U}$ is the time average of $U(t)$ and k is the discrete lag (in AIMD steps). The decay rate of $\rho(k)$ quantifies the dynamical memory of the system: a slow decay is characteristic of harmonic, solid-like motion, whereas a rapid decay signals anharmonicity, structural rearrangement or diffusive dynamics.

We estimated the (pseudo) heat capacity from energy fluctuations in a canonical-like protocol by binning frames according to instantaneous ionic temperature $T(t)$ and evaluating:

$$C_V(T) \simeq \frac{\langle U^2\rangle_T - \langle U\rangle_T^2}{k_B T^2},$$

where the averages are restricted to frames with instantaneous temperature within the bin. The melting temperature T_{melting} was taken as the temperature where one or more of the following criteria are simultaneously satisfied: (i) pronounced increase in $C_V(T)$ (local maximum), (ii) onset of sustained diffusive behaviour in the mean-squared displacement (MSD), and (iii) abrupt change in structural order parameters (ECN or first-RDF peak height). The MSD is computed as

$$\text{MSD}(t) = \frac{1}{N}\sum_{i=1}^{N}\left\langle \left|\mathbf{r}_i(t) - \mathbf{r}_i(0)\right|^2 \right\rangle,$$

and the diffusion regime is identified by a sustained, approximately linear growth of $\text{MSD}(t)$ with time.

II Density of States

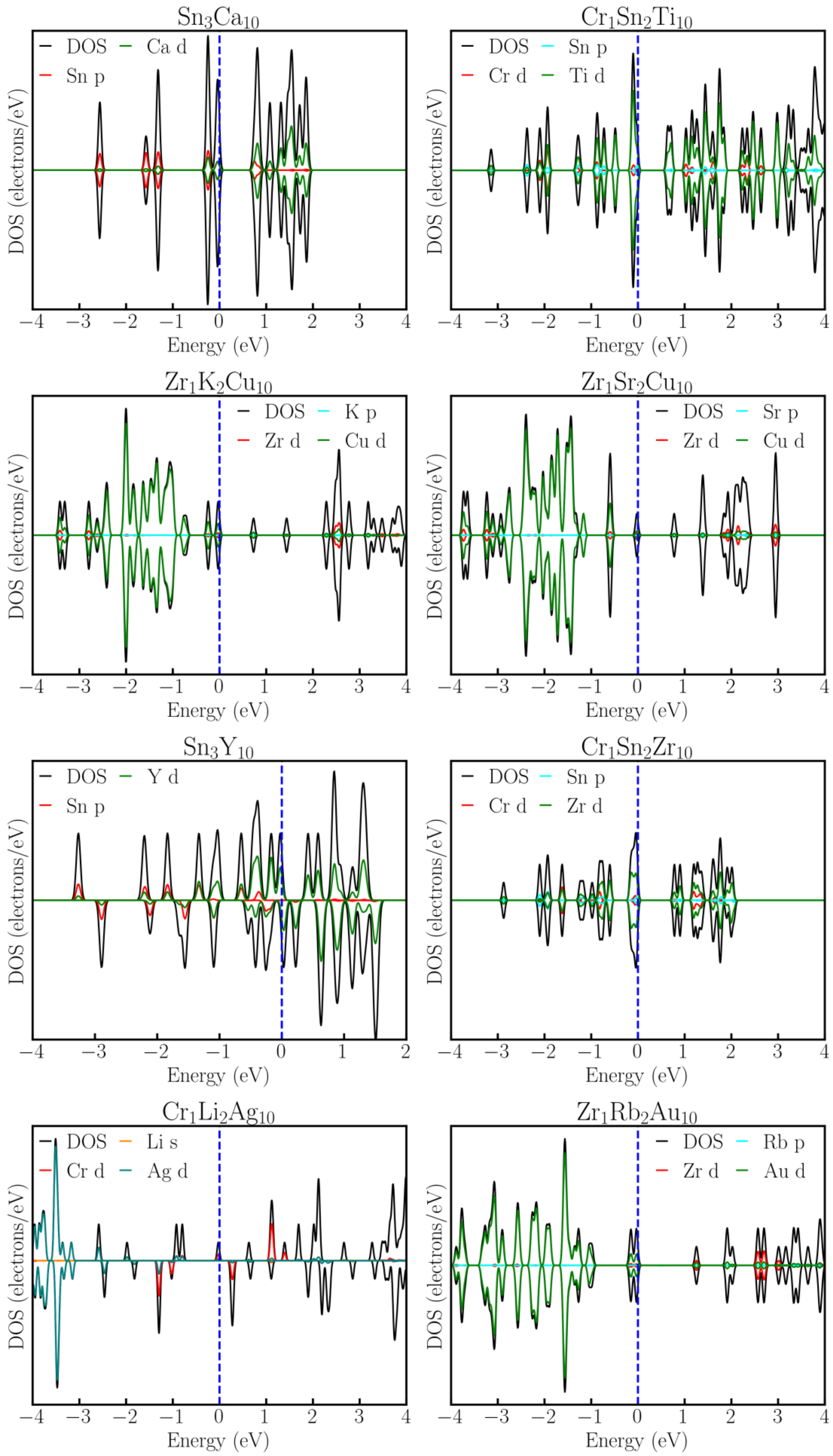

Figure S1: Electronic Density of States (DOS) for all studied clusters. The Fermi level is presented by dashed line at 0 eV.

III Electron Localization Function

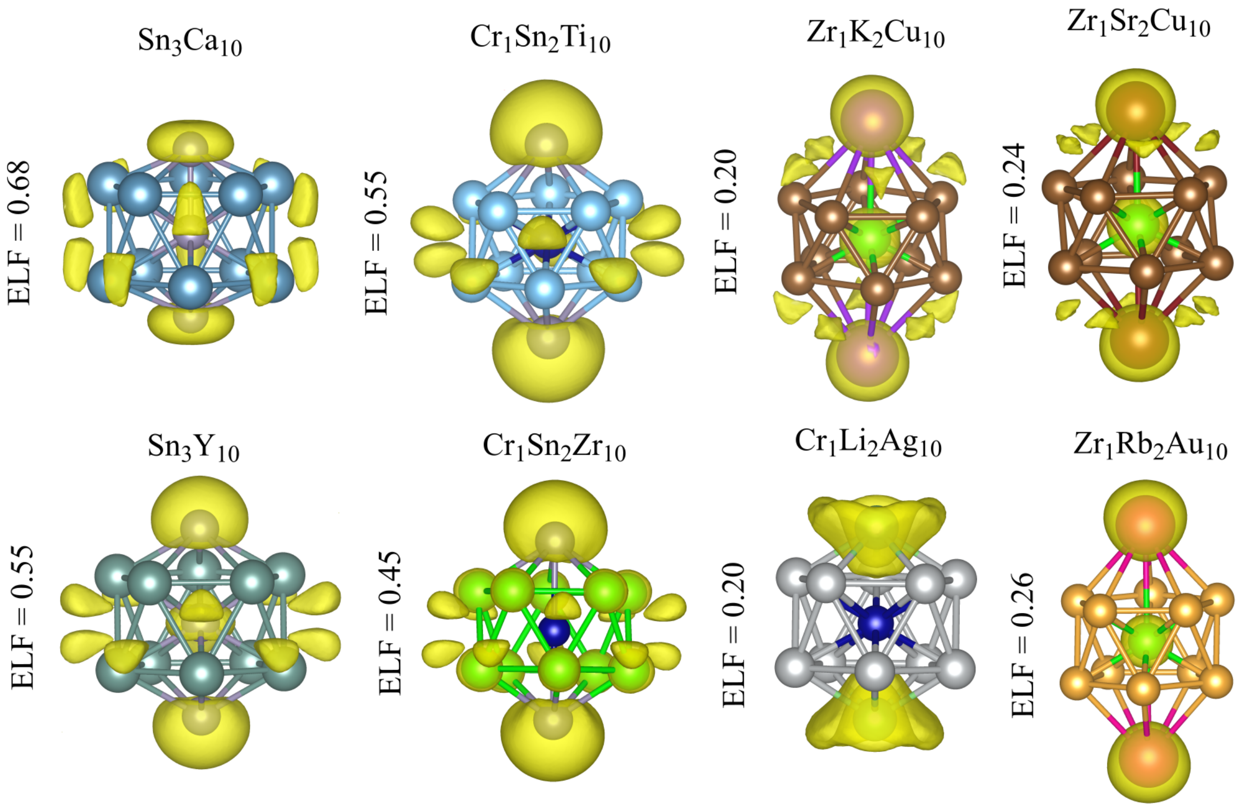

Figure S2: The electron localization function (ELF) for all studied clusters.

IV Thermalization – AIMD

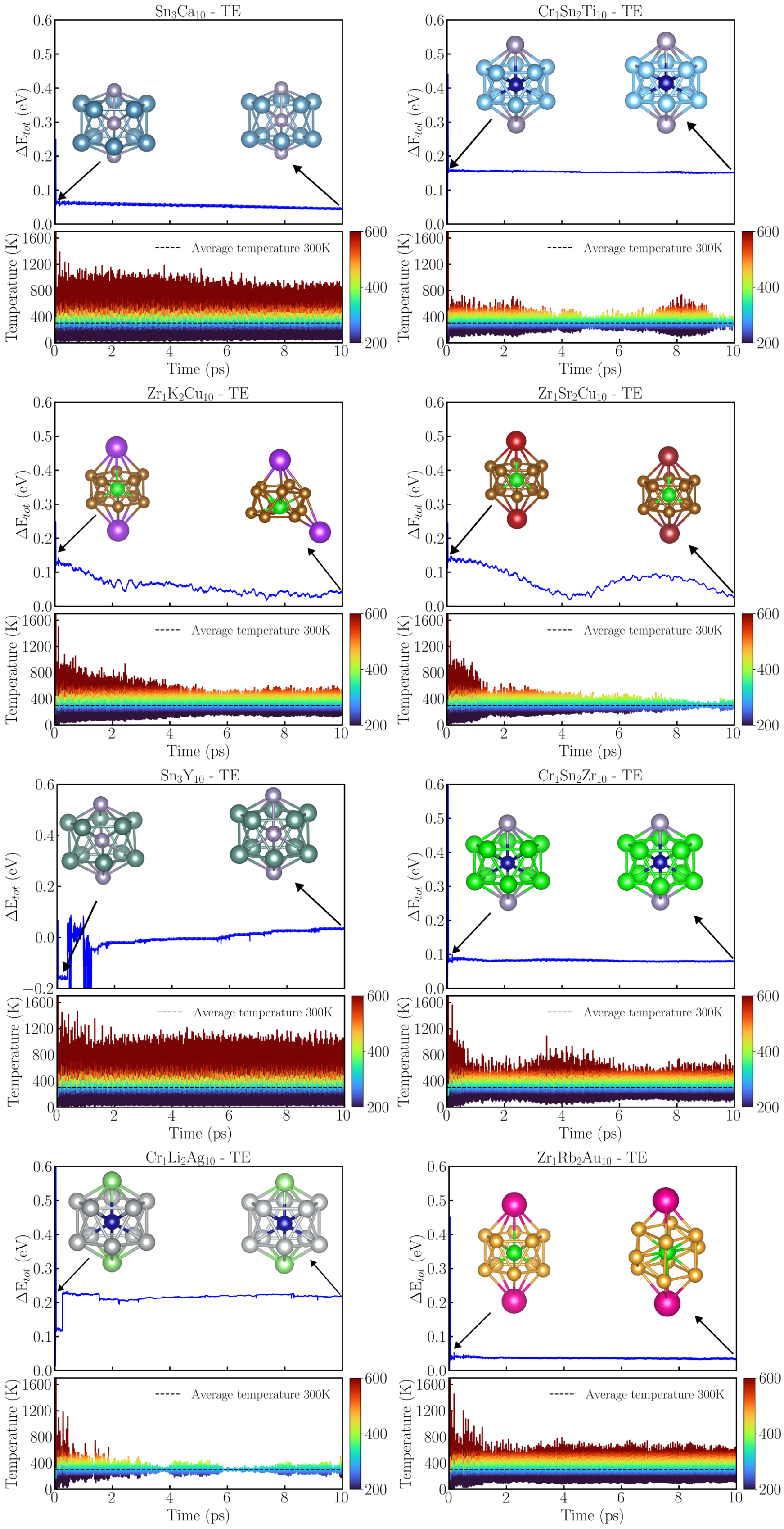

Figure S3: Thermalization at 300 K over 10 ps for all systems. Initial and final geometries are shown. The color scale represents the instantaneous ionic temperature $T(t)$, which fluctuates around the thermostat set point due to finite-size and dynamical effects inherent to small clusters.

V Melting Behavior

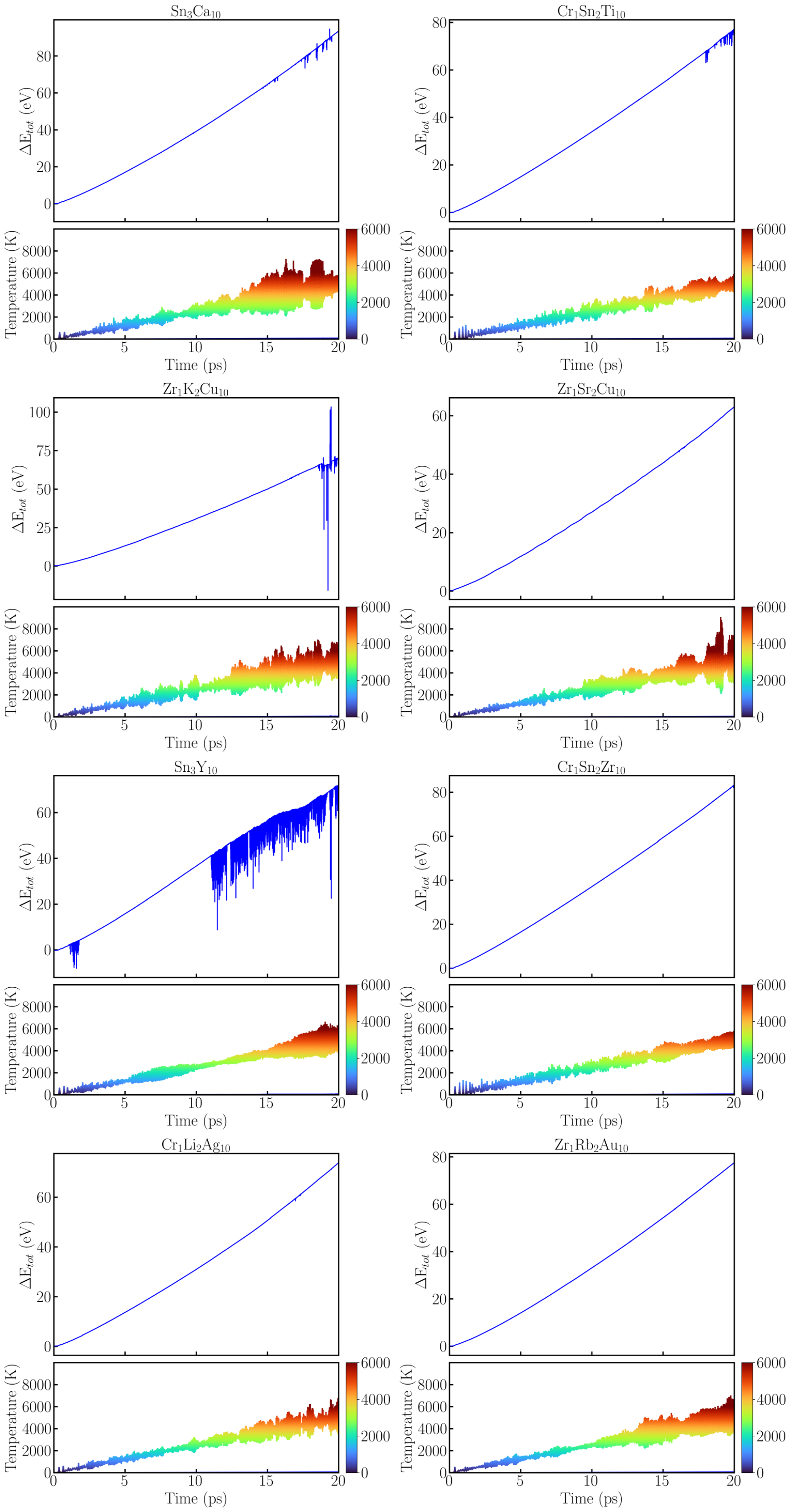

Figure S4: Simulated melting dynamics or isomerization process: annealing from 0 K to 5000 K over 20 ps.

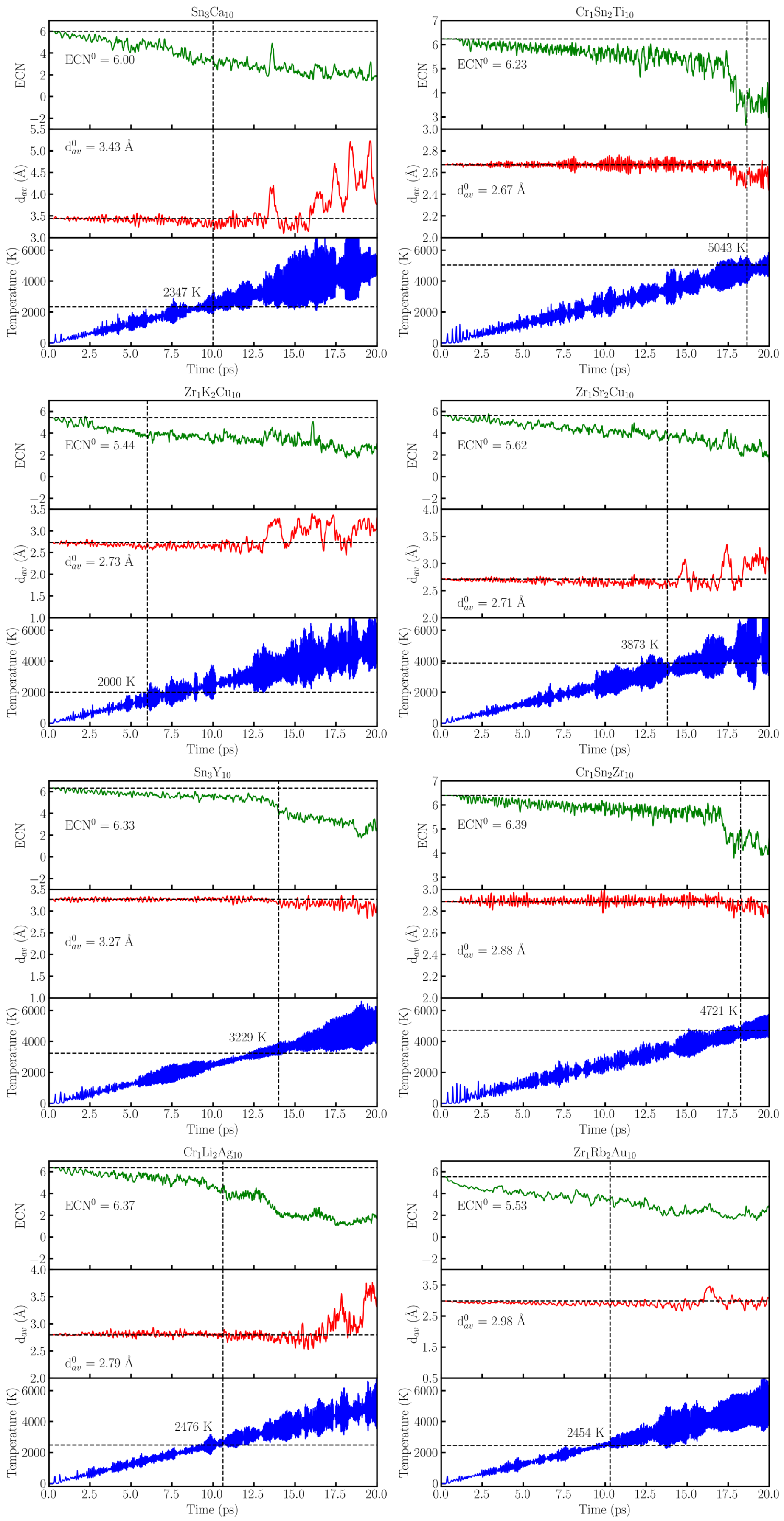

Figure S5: Evolution of structural descriptors: ECN and d_{av}, for all clusters during heating (up to 5000 K). The melting temperature (T_{melting}) is identified as the point of abrupt change in these parameters, indicating loss of structural order.

VI Potential Energy Estimation

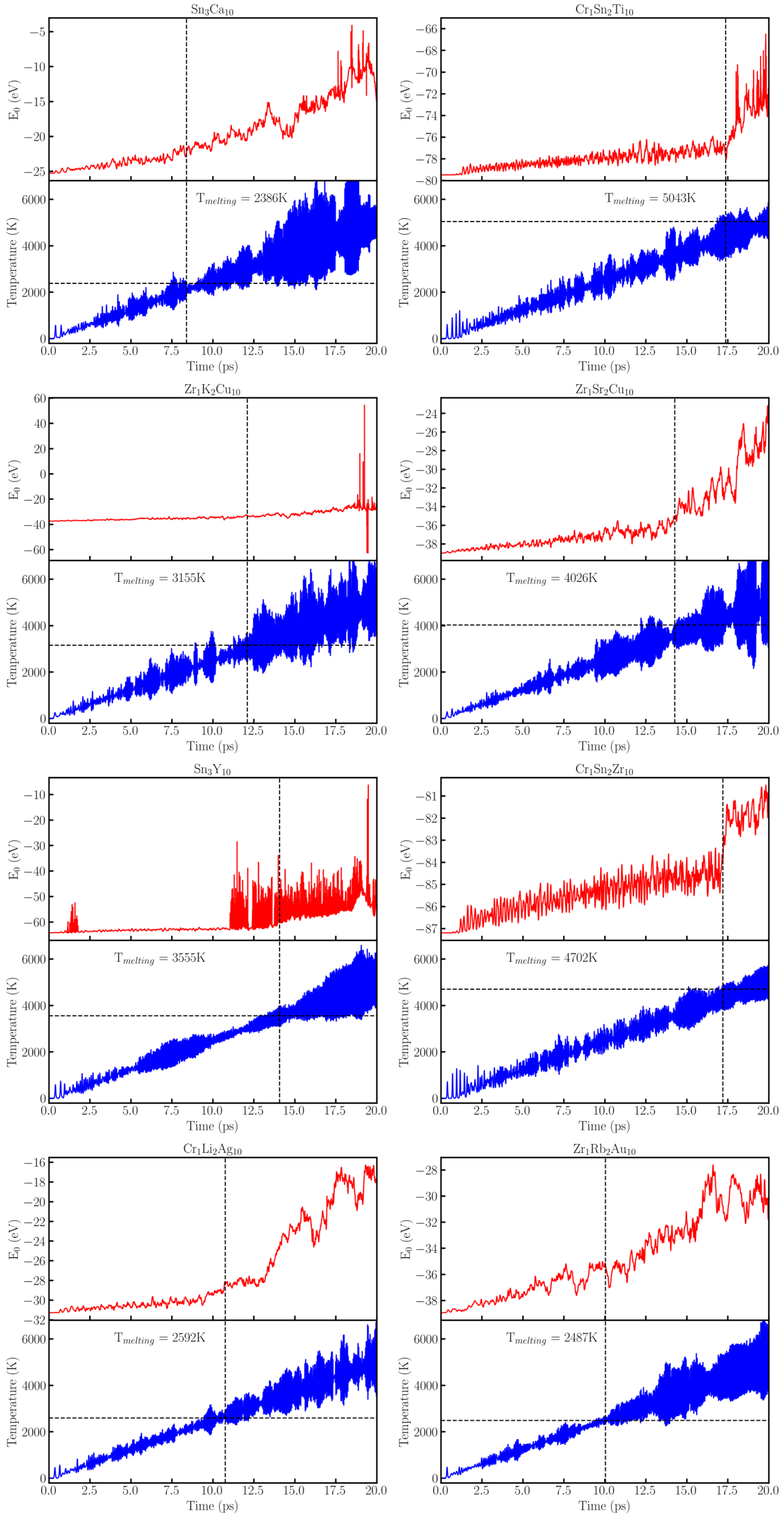

Figure S6: The instantaneous potential energy, E_0, and temperature, as functions of the time steps. The T_{melting} values are indicated by vertical traced lines.

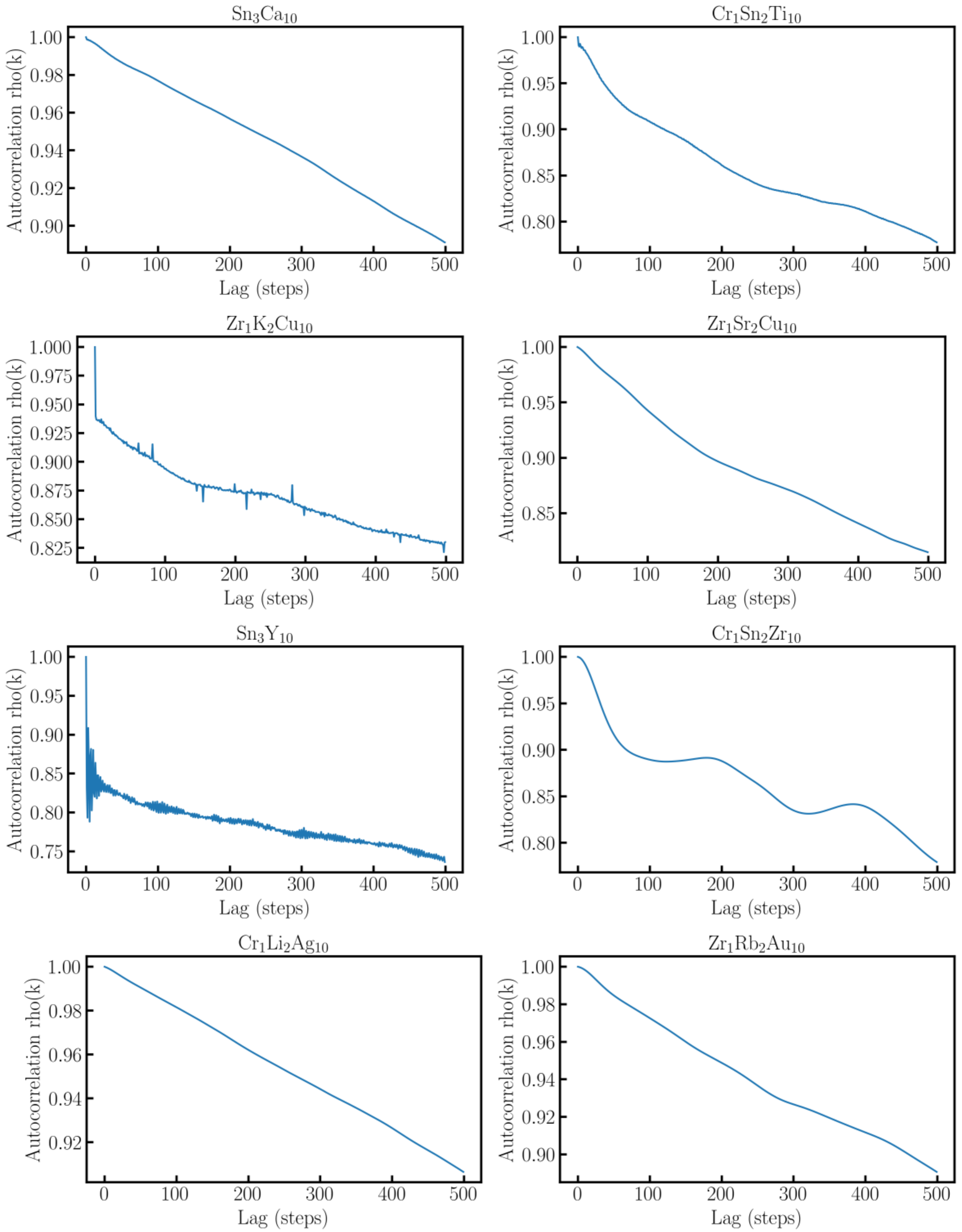

Figure S7: Energy autocorrelation function $\rho(k)$ computed from the instantaneous potential energy $E_0(t)$ for all clusters. Slow decay of $\rho(k)$ indicates persistent vibrational memory and solid-like dynamics; rapid decay denotes anharmonicity and loss of structural coherence (softening or onset of diffusion).

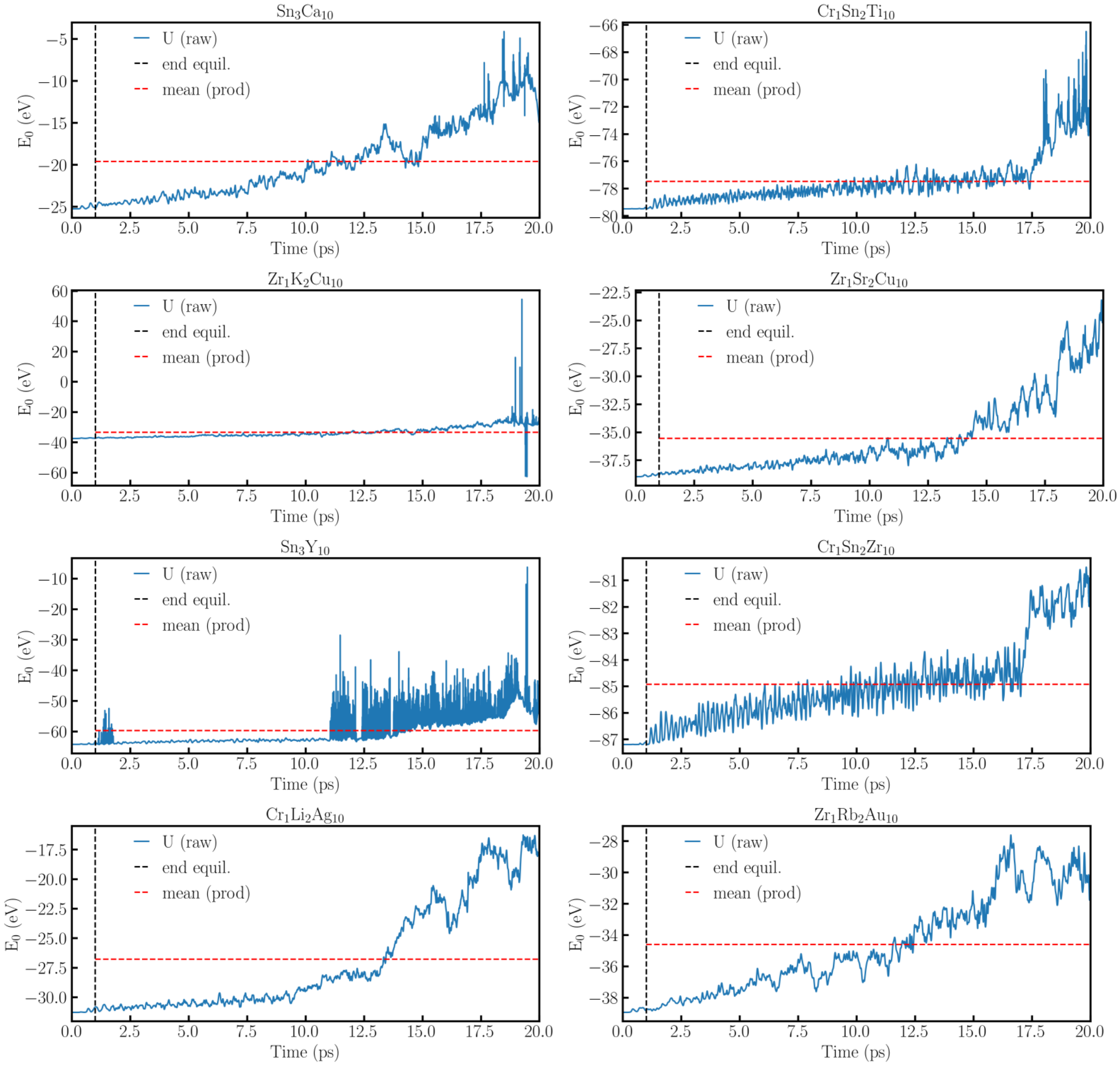

Figure S8: Instantaneous potential energy $E_0(t)$ trajectories for the studied clusters. Stable clusters show small fluctuations; unstable clusters show large fluctuations and baseline shifts.

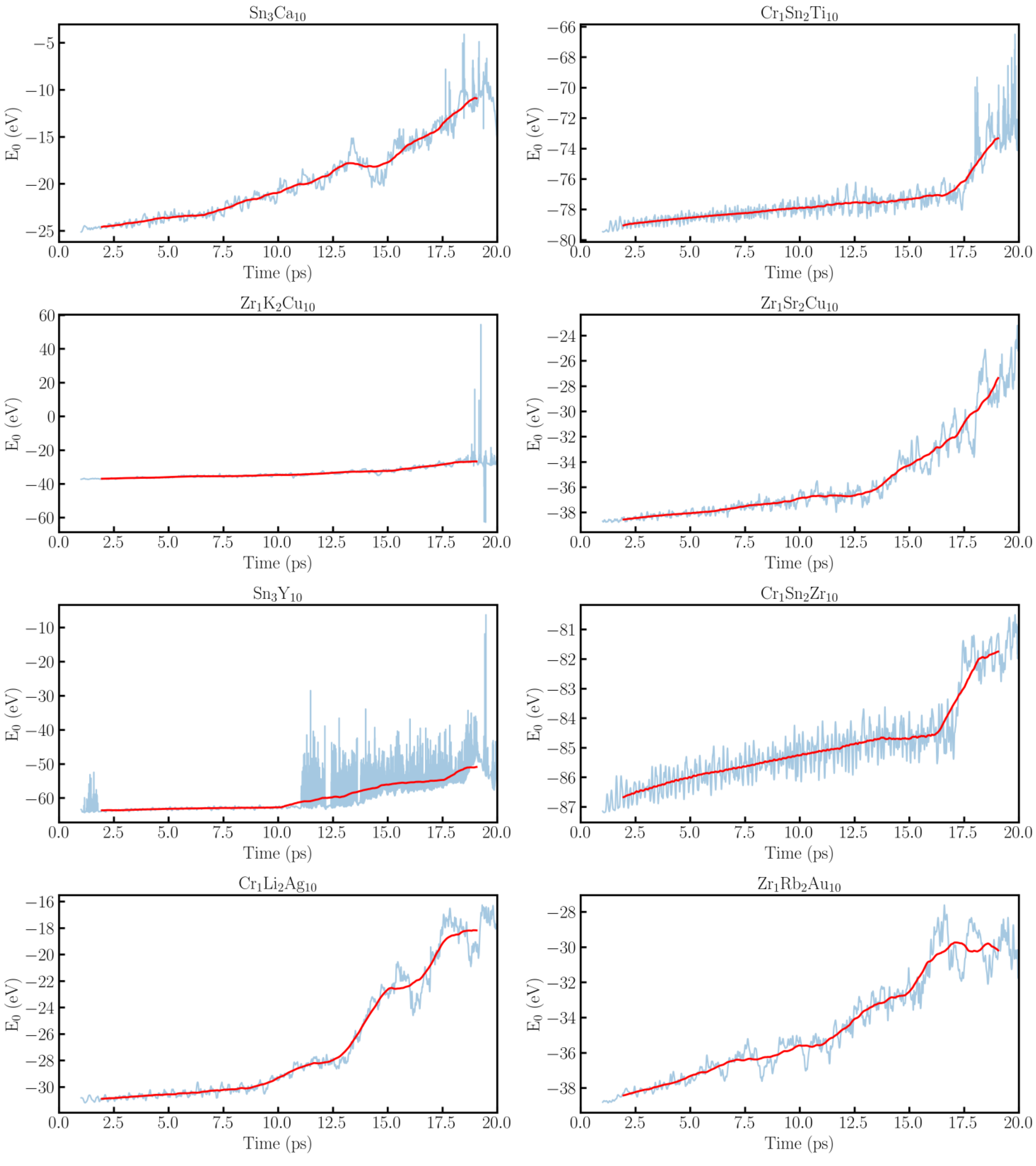

Figure S9: Instantaneous potential energy $E_0(t)$ trajectories for all clusters, highlighting the contrast in thermal stability.

VII Melting Indicators for Individual Clusters from Parallel Tempering Simulations

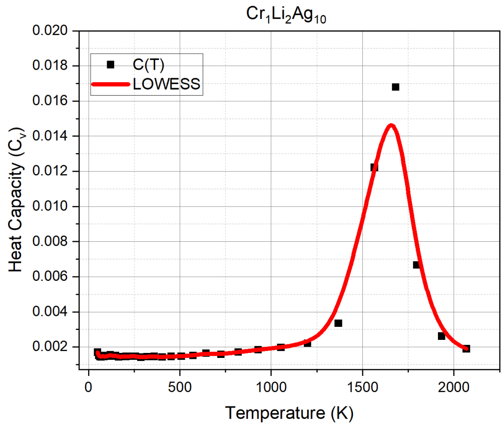

(a) Heat capacity $(C(T))$ curve.

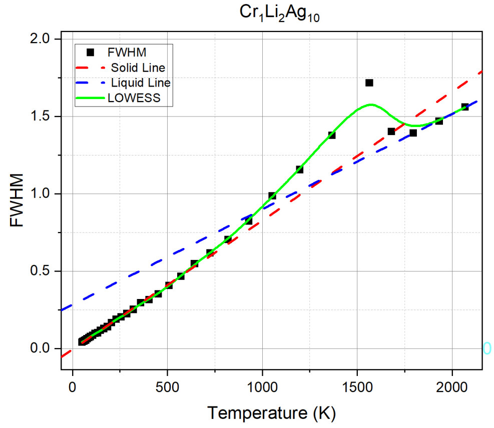

(b) Width of the potential energy distribution. (W_U)

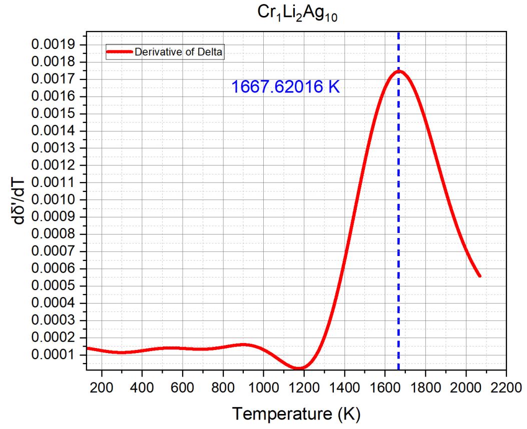

(c) Derivative of δ with respect to temperature.

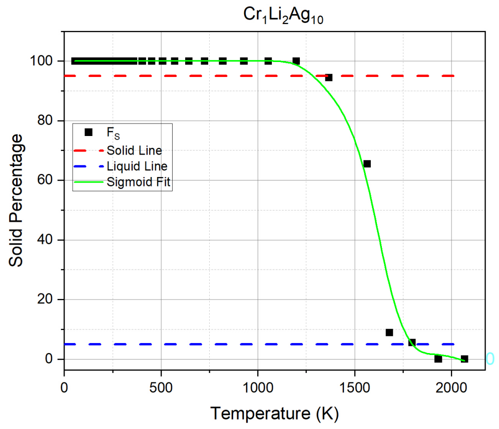

(d) Percentage of configurations classified as solid.(ANNC)

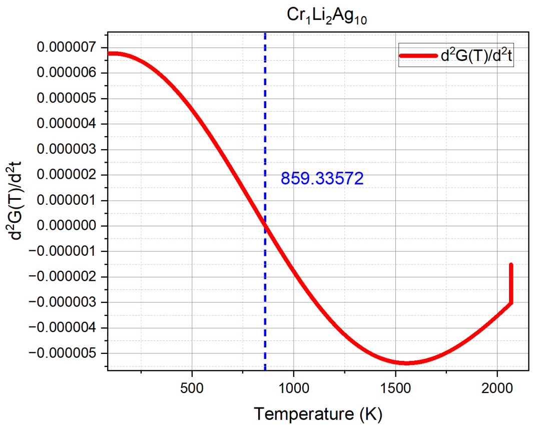

(e) Second derivative of G with respect to temperature (G)

Figure S10:Temperature dependence of properties used to detect melting for $CrLi_2Ag_{10}$: $C(T), W_U, \delta$, ANNC F_S, and inflection point d^2G/dT^2.

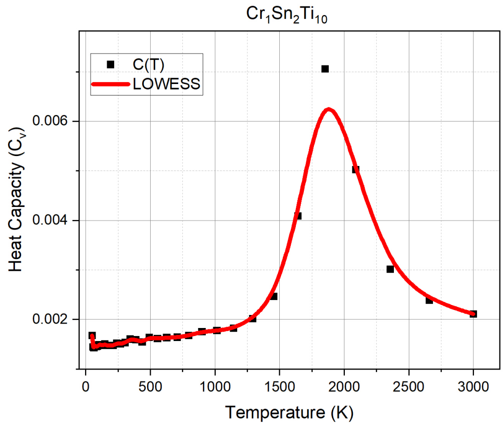

(a) Heat capacity $(C(T))$ curve.

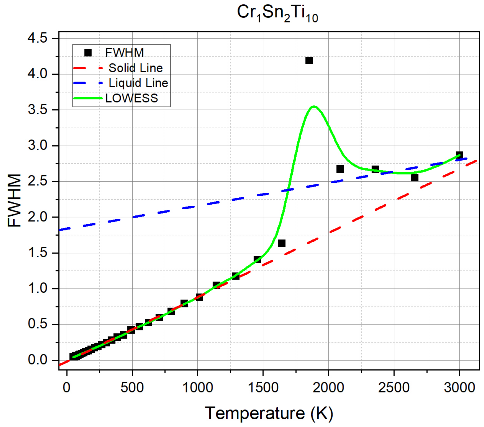

(b) Width of the potential energy distribution. (W_U)

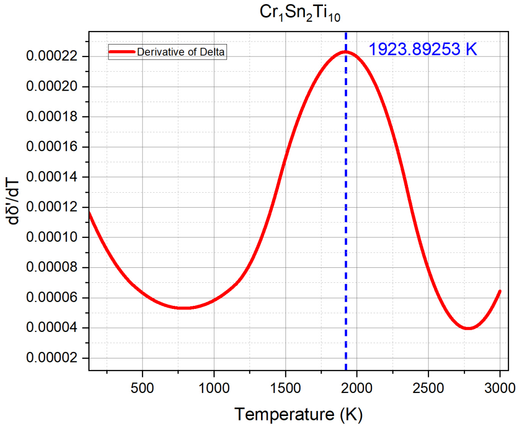

(c) Derivative of δ with respect to temperature.

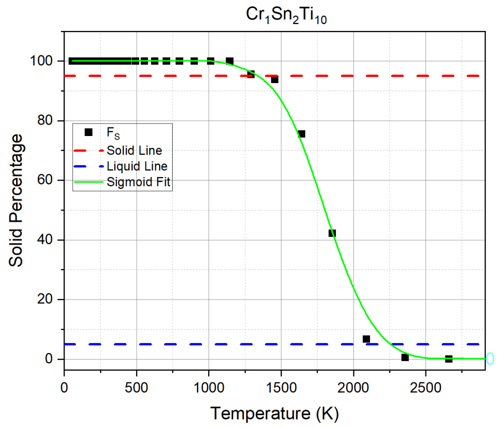

(d) Percentage of configurations classified as solid.(ANNC)

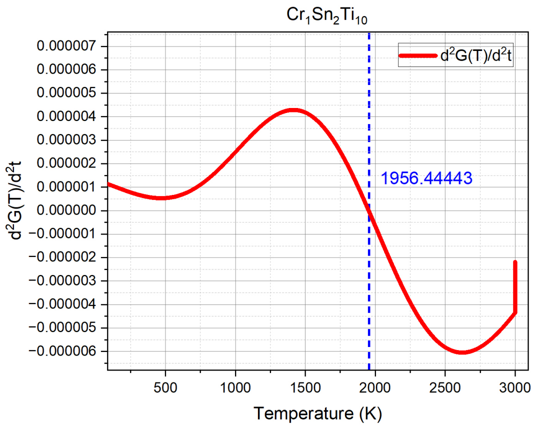

(e) Second derivative of G with respect to temperature (G)

Figure S11:Temperature dependence of properties used to detect melting for $CrSn_2Ti_{10}$: $C(T)$, W_U, δ, ANNC F_S, and inflection point d^2G/dT^2.

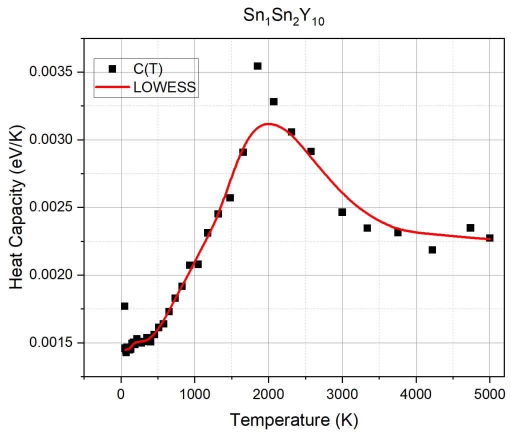

(a) Heat capacity $(C(T))$ curve.

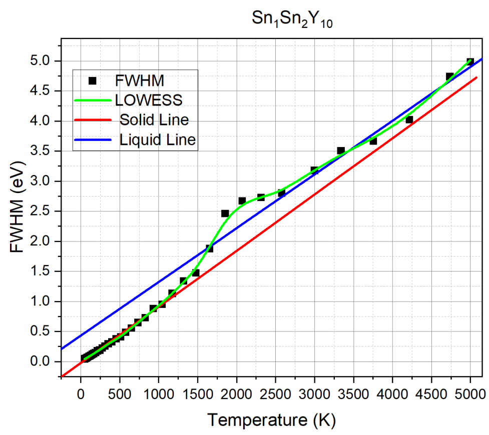

(b) Width of the potential energy distribution. (W_U)

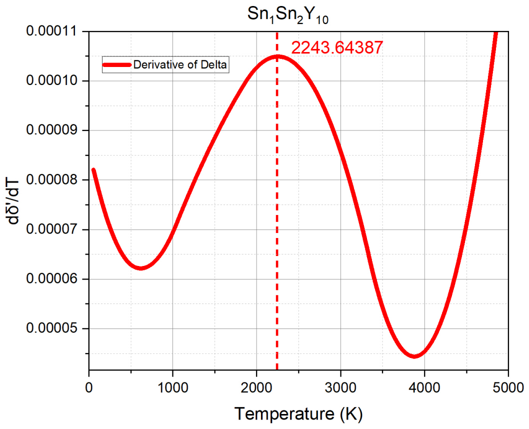

(c) Derivative of δ with respect to temperature.

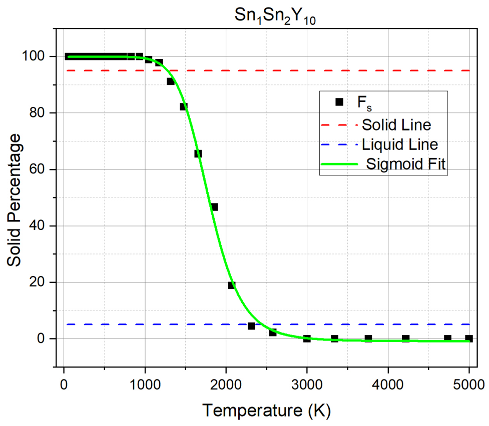

(d) Percentage of configurations classified as solid.(ANNC)

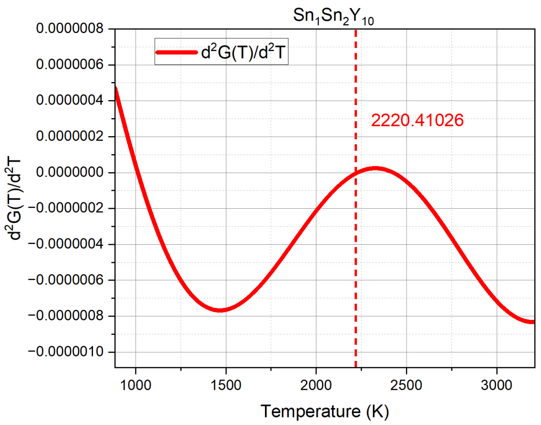

(e) Second derivative of G with respect to temperature (G)

Figure S12:Temperature dependence of properties used to detect melting for Sn_3Y_{10} : $C(T)$, W_U, δ, ANNC F_S, and inflection point d^2G/dT^2.

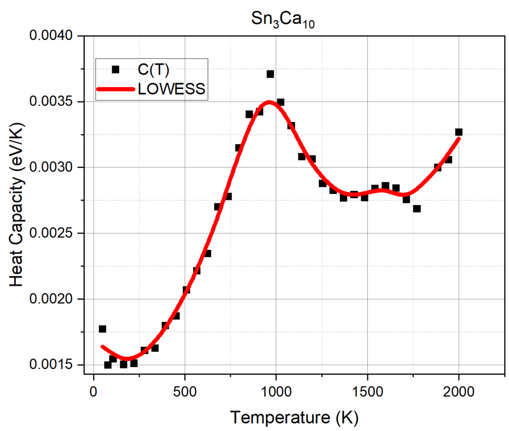

(a) Heat capacity $(C(T))$ curve.

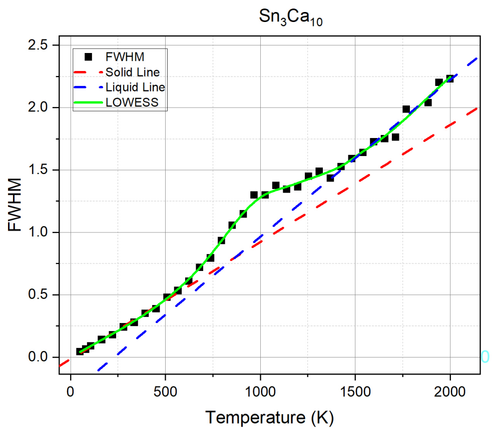

(b) Width of the potential energy distribution. (W_U)

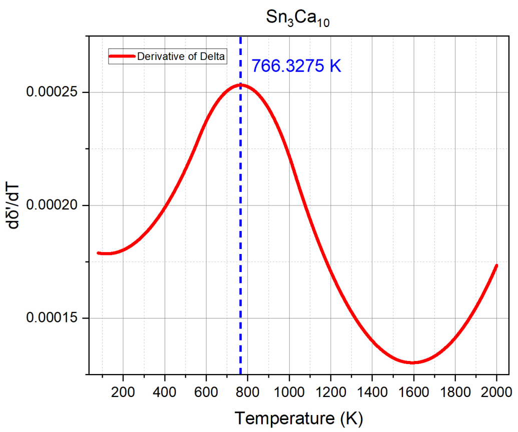

(c) Derivative of δ with respect to temperature.

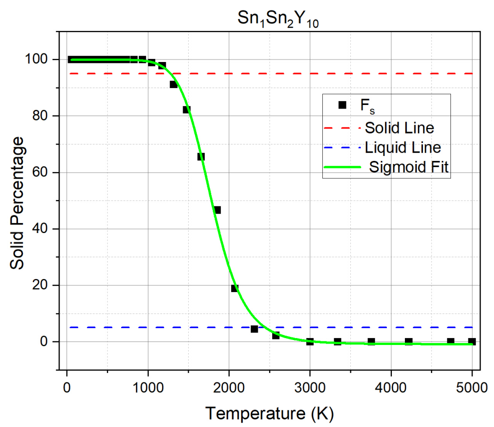

(d) Percentage of configurations classified as solid.(ANNC)

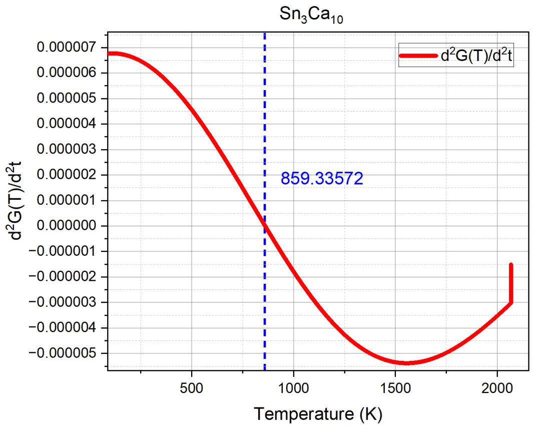

(e) Second derivative of G with respect to temperature (G)

Figure S13:Temperature dependence of properties used to detect melting for Sn_3Ca_{10} : $C(T)$, W_U, δ, ANNC F_S, and inflection point d^2G/dT^2.

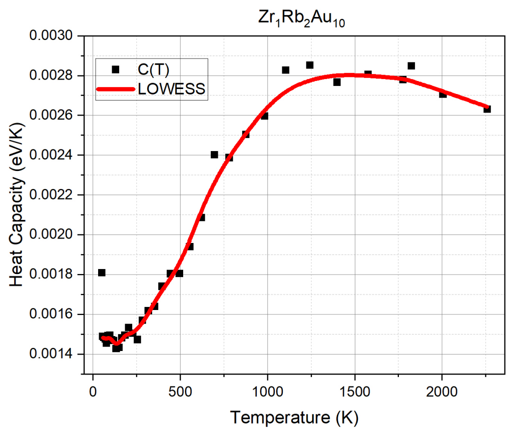

(a) Heat capacity $(C(T))$ curve.

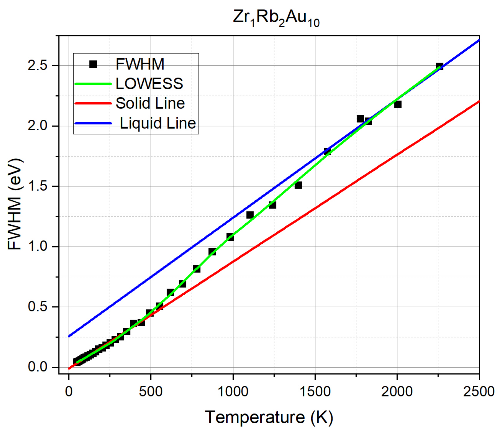

(b) Width of the potential energy distribution. (W_U)

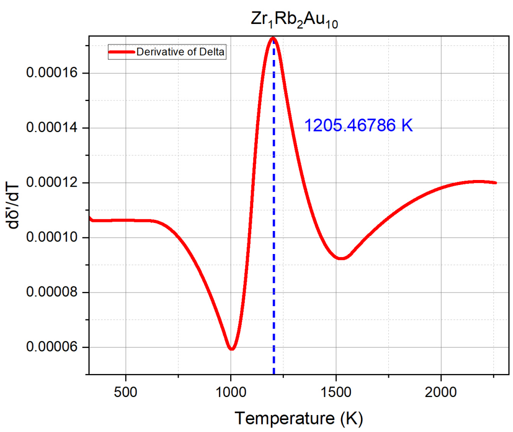

(c) Derivative of δ with respect to temperature.

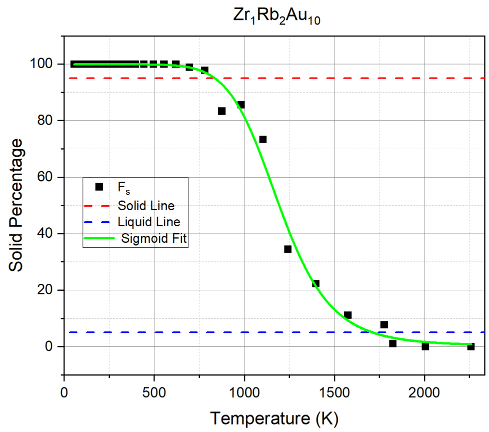

(d) Percentage of configurations classified as solid.(ANNC)

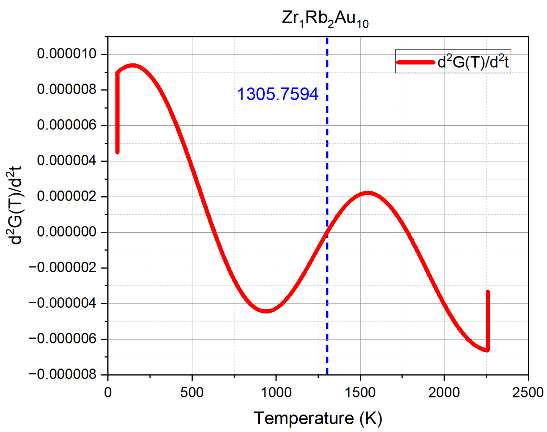

(e) Second derivative of G with respect to temperature (G)

Figure S14:Temperature dependence of properties used to detect melting for $ZrRb_2Au_{10}$: $C(T), W_U, \delta$, ANNC F_S, and inflection point d^2G/dT^2.

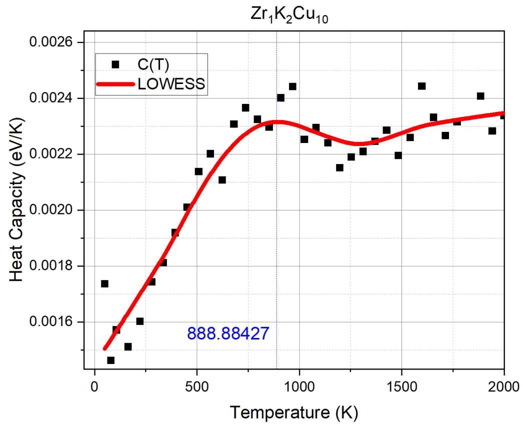

(a) Heat capacity $(C(T))$ curve.

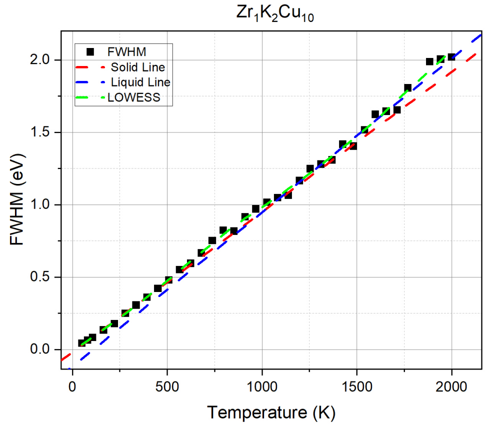

(b) Width of the potential energy distribution. (W_U)

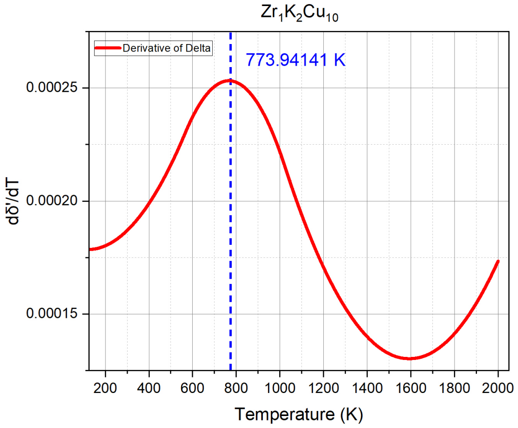

(c) Derivative of δ with respect to temperature.

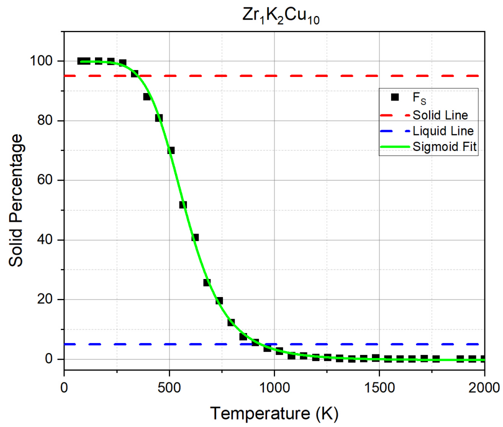

(d) Percentage of configurations classified as solid.(ANNC)

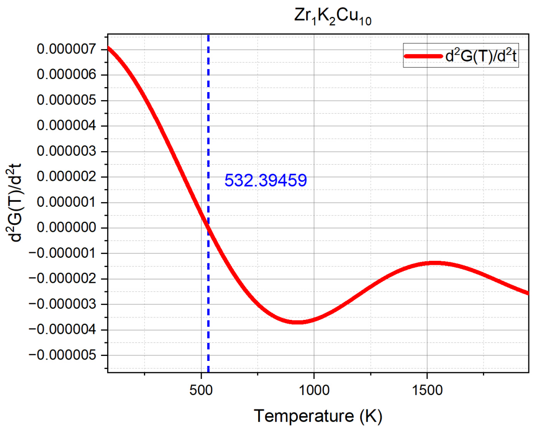

(e) Second derivative of G with respect to temperature (G)

Figure S15:Temperature dependence of properties used to detect melting for ZrK_2Cu_{10} : $C(T)$, W_U, δ, ANNC F_S, and inflection point d^2G/dT^2.

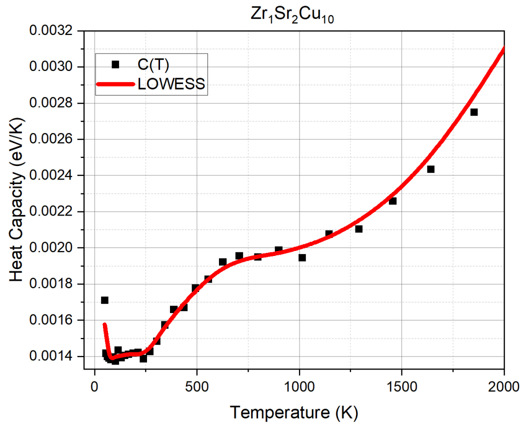

(a) Heat capacity $(C(T))$ curve.

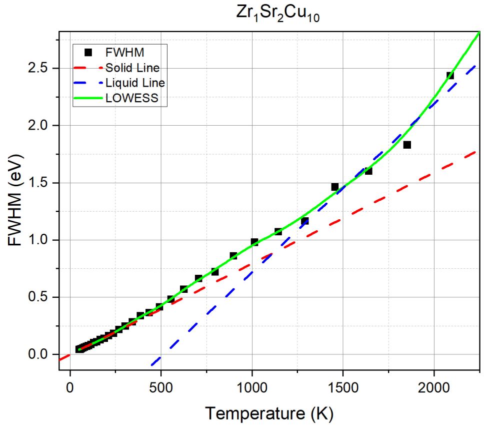

(b) Width of the potential energy distribution. (W_U)

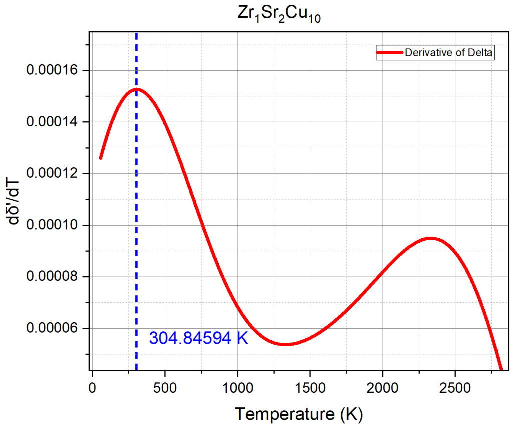

(c) Derivative of δ with respect to temperature.

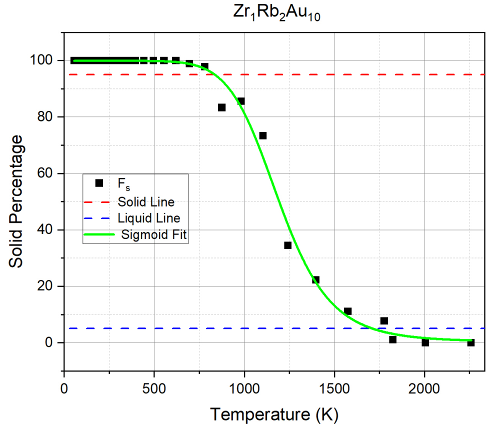

(d) Percentage of configurations classified as solid.(ANNC)

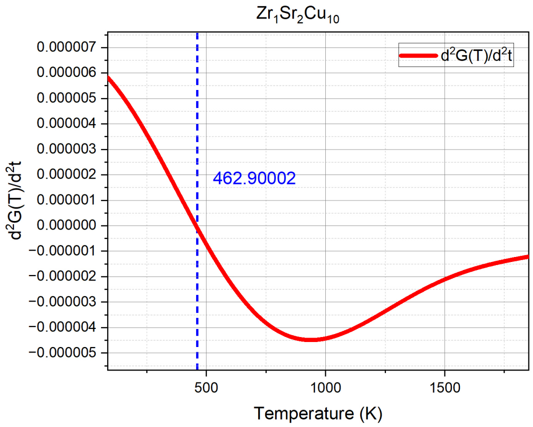

(e) Second derivative of G with respect to temperature (G)

Figure S16:Temperature dependence of properties used to detect melting for $ZrSr_2Cu_{10}$: $C(T), W_U, \delta$, ANNC F_S, and inflection point d^2G/dT^2.
